# FET fusion oncoproteins enrich SWI/SNF complex subtypes and interaction partners

**DOI:** 10.1186/s11658-025-00792-w

**Published:** 2025-09-23

**Authors:** Malin Lindén, Lisa Andersson, Heba Albatrok, Vilma Canfjorden, Emma Jonasson, Kajsa Grönqvist, Daniel Sjövall, Pekka Jaako, Rossella Crescitelli, Henrik Fagman, Pierre Åman, Anders Ståhlberg

**Affiliations:** 1 Department of Laboratory Medicine, Institute of Biomedicine, Sahlgrenska Center for Cancer Research, Sahlgrenska Academy, University of Gothenburg, Gothenburg, Sweden; 2 Department of Microbiology and Immunology, Institute of Biomedicine, Sahlgrenska Center for Cancer Research, Sahlgrenska Academy, University of Gothenburg, Gothenburg, Sweden; 3 Department of Surgery, Institute of Clinical Sciences, Sahlgrenska Center for Cancer Research, Sahlgrenska Academy, University of Gothenburg, Gothenburg, Sweden; 4 Wallenberg Centre for Molecular and Translational Medicine, University of Gothenburg, Gothenburg, Sweden; 5 Department of Clinical Genetics and Genomics, Sahlgrenska University Hospital, Gothenburg, Region Västra Götaland, Sweden; 6 Science for Life Laboratory, Institute of Biomedicine, University of Gothenburg, Gothenburg, Sweden

**Keywords:** Ewing sarcoma, FET fusion oncoproteins, Myxoid liposarcoma, Quantitative mass spectrometry, SWI/SNF chromatin remodeling complex

## Abstract

**Background:**

FET (FUS, EWSR1, and TAF15) fusion oncoproteins are characteristic for several sarcomas and leukemias, including myxoid liposarcoma and Ewing sarcoma. FET oncoproteins interact with the SWI/SNF chromatin remodeling complex subtypes cBAF, PBAF, and GBAF, but their impact on SWI/SNF compositions, interactions, and downstream epigenetic effects remains elusive.

**Methods:**

We employ a comprehensive immunoprecipitation and quantitative mass spectrometry approach to determine the impact of FET oncoproteins on SWI/SNF composition and their interactomes. Validation of complex composition and interaction partners is performed by glycerol gradient sedimentation assays and co-immunofluorescence analysis. Furthermore, we determine the differential chromatin accessibility and gene regulation in FET sarcomas using assay for transposase-accessible chromatin sequencing and RNA sequencing, respectively.

**Results:**

Our data show that FET sarcomas have distinct SWI/SNF complex compositions, with different subunit paralogs and subtype-specific components that utilize distinct sets of interaction partners, including specific transcription factors. We show that FET oncoproteins cause no major disruption of the SWI/SNF complex composition. Instead, FUS::DDIT3-bound SWI/SNF complexes in myxoid liposarcoma cells are enriched in PBAF and GBAF components as well as most interaction partners.

**Conclusions:**

These data suggest that FET oncoproteins act together with fully assembled and functional SWI/SNF complexes and recruited interaction partners. Finally, our data reveal that the SWI/SNF compositions, interactomes, and epigenetic background contribute to the tumor type in FET sarcoma.

*Trial registration* Clinical trial number: not applicable.

**Graphical Abstract:**

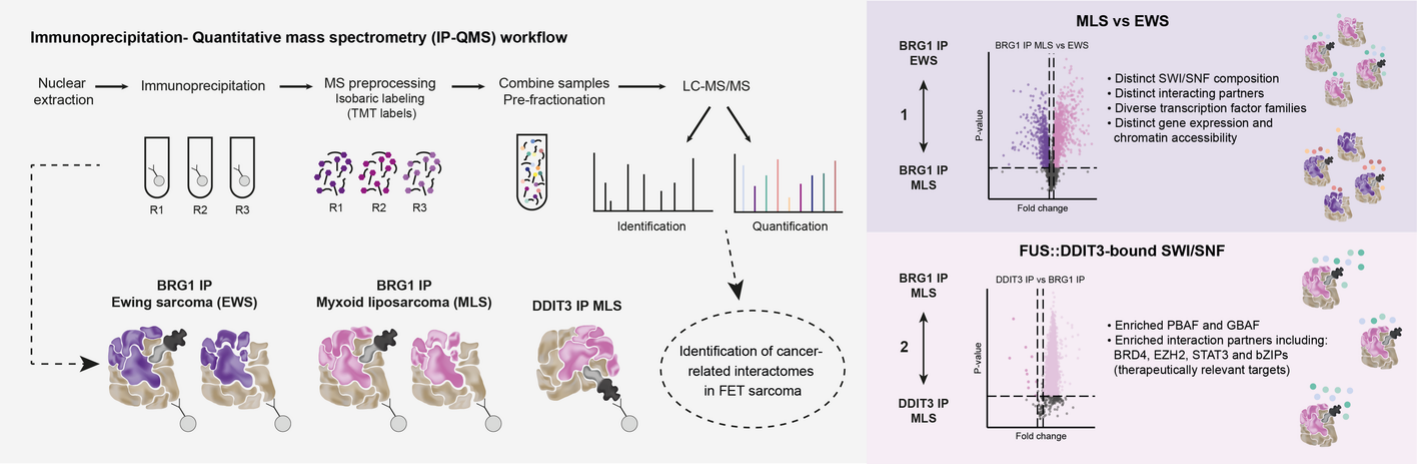

**Supplementary Information:**

The online version contains supplementary material available at 10.1186/s11658-025-00792-w.

## Background

**Fig. 1:**
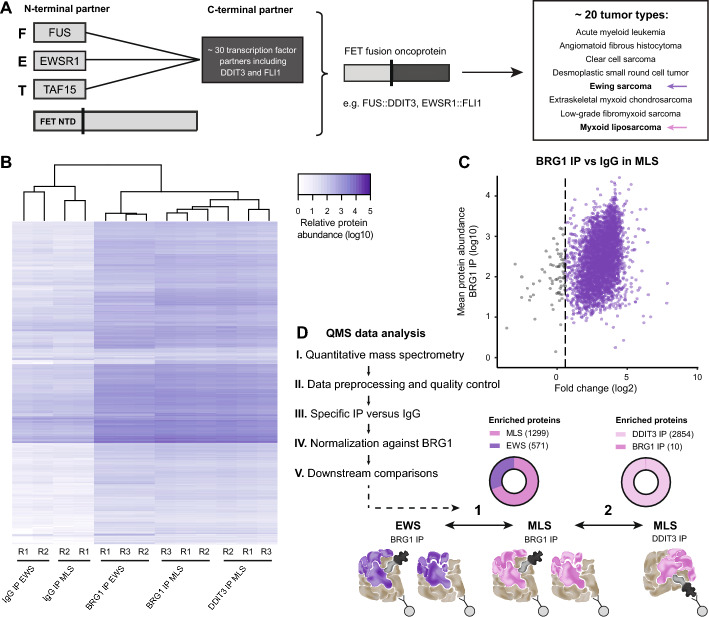
Strategy to identify specific SWI/SNF interactors using IP-QMS in FET sarcoma. **A** Schematic visualization of FET fusion oncoproteins containing an N-terminal FET protein (FUS, EWSR1, or TAF15 N-terminal domain, NTD) and a C-terminal transcription-factor partner formed after a chromosomal translocation. FET oncoproteins are characteristic for specific tumor entities, including Ewing sarcoma and myxoid liposarcoma. A few examples are shown. **B** Heatmap of relative protein abundances in IP samples: IgG IP in EWS and MLS, BRG1 IP in EWS and MLS, and DDIT3 IP in MLS. **C** Scatter plot showing the mean abundance of proteins in BRG1 IP versus the enrichment of proteins in BRG1 IP versus IgG in MLS (*n* = 3131). Each dot indicates a protein, where non-enriched (gray) and enriched (purple) proteins are marked, dashed lines indicate the applied fold change cutoff of 1.5. **D** Workflow of QMS data analysis and schematic visualization of the two downstream comparisons (1) BRG1 IP in MLS versus BRG1 IP in EWS and (2) DDIT3 IP in MLS versus BRG1 IP in MLS. The number and fraction of significant enriched proteins are visualized in the pie charts using fold change > 1.2 and *p*-value < 0.05 as cutoff values

The FET (*FUS*, *EWSR1*, and *TAF15*) fusion oncogenes are characteristic for around 20 different sarcomas and leukemia, including *FUS*::*DDIT3* in myxoid liposarcoma (MLS) and *EWSR1*::*FLI1* in Ewing sarcoma (EWS), the two most common FET sarcoma entities (Fig. 1A). All FET fusion oncoproteins (FET-FOPs) contain the N-terminal part of one of the FET proteins, consisting of intrinsically disordered regions (IDR) rich in SYGQ repeats [1]. These prion-like regions enable protein–protein interactions, occasionally leading to liquid–liquid phase separation and formation of biocondensates [2]. The C-terminal of FET-FOPs consists of parts of one of many alternative transcription factors that contributes with DNA-binding capacity. FET oncoproteins induce oncogenic gene expression profiles by acting as aberrant transcription factors [3–5]. However, the tumor-type-specific properties of FET-FOPs indicate that oncogenesis is limited to certain contexts, such as specific cell types or developmental stages, with a permitting epigenetic landscape [6]. More research is required to delineate the link between the function of FET oncoproteins and the epigenetic background.

The SWI/SNF chromatin remodeling complex is a major interaction partner of FET oncoproteins [7–9]. SWI/SNF complexes remodel nucleosomes and expose DNA by utilizing the energy from ATP hydrolysis [10]. By remodeling the nucleosome landscape, SWI/SNF complexes directly affect transcription, replication, and repair. Furthermore, they affect cellular processes, such as differentiation and proliferation, via interactions with specific transcription factors [11]. Some transcription factors can act as pioneering factors, guiding the SWI/SNF complex to distinct sites resulting in altered chromatin accessibility while others are dependent on the action of chromatin modifying complexes, thereafter binding to newly accessible regions [12–16]. SWI/SNF complexes consist of around 10–15 subunits encoded by 29 genes, including the ATPase BRG1. Some subunits exist as different paralogs, and their combinatorial assembly results in a large variety of distinct complexes associated with specific functions, for example in tissue development [17]. Recently, three main subtypes of the SWI/SNF complex were described: the canonical cBAF complex, the PBAF complex, and the GBAF complex (also called ncBAF) [18, 19]. The subtypes display both shared and distinct genome binding patterns and functions owing to the contributions of specific subunits [20–22]. SWI/SNF components are mutated in around 20% of all human tumors, demonstrating a key role in tumorigenesis [23]. Although these are commonly loss-of-function mutations, a large part of the oncogenic role is attributed to the abnormal activity of residual SWI/SNF complexes [11, 24]. Normally, FET sarcomas have no mutations in SWI/SNF genes. Instead, the binding of FET-FOPs to SWI/SNF complexes results in aberrant SWI/SNF composition and function, such as genomic targeting and opposition of the Polycomb repressor complex 2 (PRC2) [7, 8, 25, 26]. In MLS, SWI/SNF complexes can be redistributed by FUS::DDIT3 from adipogenic promotor regions [26], while in EWS, they can be aberrantly recruited by EWSR1::FLI1 to tumor-specific enhancers [8]. Nevertheless, the exact role of aberrant SWI/SNF complexes and their compositions in FET sarcoma remain to be elucidated.

The aim of the present study was to determine the impact of FET oncoproteins on SWI/SNF composition and interactomes in MLS and EWS to delineate their specific oncogenic roles in FET sarcoma. We employed a strategy involving immunoprecipitation (IP) and quantitative mass spectrometry (QMS) analysis, either pulling down all SWI/SNF complexes using BRG1 as a target or FET-FOP-bound SWI/SNF complexes using the oncoprotein as a target. This allowed us to assess differences between SWI/SNF composition and interaction partners in MLS and EWS cells, as well as determine the composition and interactome of FET-FOP-bound SWI/SNF complexes. SWI/SNF subtypes and selected interaction partners were validated with glycerol gradient sedimentation assays and co-immunofluorescence analysis. Furthermore, we evaluated the differential chromatin accessibility and gene regulation using assay for transposase-accessible chromatin sequencing (ATAC-seq) and RNA sequencing (RNA-seq) to discern the epigenetic landscapes in FET sarcoma.

## Methods

All detailed methods are included in the main article or as supplementary data (Supplementary Methods, Additional file 1).

### Cell culture

Myxoid liposarcoma cell lines MLS 1765–92 (RRID:CVCL_S817), 402–91 (RRID:CVCL_S813) [27], and 2645–94 (RRID:CVCL_S816; gift from Dr B. Kazmierczak, Bremen, Germany) [28] containing FUS::DDIT3 type 13–2, type I/7–2 and type II/5–2, respectively, Ewing sarcoma cell lines (gift from Dr. Katia Scotlandi, University of Bologna, Italy) EWS TC-71 (RRID:CVCL_2213) and EWS 6647 (RRID:CVCL_H722) containing EWSR1::FLI1 type 1 and 2, respectively, and fibrosarcoma HT1080 (ATCC: CCL-121, RRID:CVCL_0317) [29] were cultured in media supplemented with 100 U/ml penicillin and 100 μg/ml streptomycin, either RPMI 1640 GlutaMAX with 5% fetal bovine serum for MLS and HT1080 or IMDM GlutaMAX with 10% fetal bovine serum for EWS cells (all Gibco, Thermo Fisher Scientific). The endometrial adenocarcinoma HEC-1-A (ATCC: HTB-112, RRID:CVCL_0293) and colorectal adenocarcinoma DLD-1 (ATCC: CCL-221, RRID:CVCL_0248) cell lines were cultured as described by ATCC. Cells were passaged using 0.25% trypsin supplemented with 0.5 mM ethylenediaminetetraacetic acid (EDTA) (Gibco) and maintained at 37 °C and 5% CO_2_. All cell lines were routinely screened for mycoplasma infections using a mycoplasma polymerase chain reaction detection kit (ScienCell Research Laboratories). MLS 402–91 and MLS 1765–92 are available upon request.

### Nuclear protein extraction

MLS 402–91 and EWS TC-71 cells from at least two 15-cm cell culture dishes (Nunc, Thermo Fisher Scientific) were harvested by scraping in Dulbecco’s phosphate-buffered saline (Gibco). Nuclear protein extraction was performed as previously described [25], except a high-salt extraction buffer with 0.87 M KCl (10 mM Tris pH 7.5, 0.1 mM EDTA; Thermo Fisher Scientific and 10% glycerol; Merck Chemicals, Merck, supplemented with 1 × protease inhibitor) was used during incubation on ice for 30 min, thereby extracting nuclear proteins and equalizing the cells to approximate 500 mM KCl. The supernatant containing the nuclear fraction was collected after centrifugation at 20,000 rcf for 5 min at 4 °C, snap-frozen on dry ice in the high-salt buffer to minimize protein aggregation, and stored at −80 °C. The DC Protein Assay (Bio-Rad Laboratories) was used to determine the protein concentration. Before analysis, thawed nuclear extract was diluted to 150 mM KCl salt concentration.

### Immunoprecipitation

Protein complexes were pulled down using a direct IP protocol suitable for downstream mass spectrometry analysis, in technical replicates. First, 37.5 µl magnetic beads (Dynabeads Myone Streptavidin T1, Thermo Fisher Scientific) per reaction were blocked in 1 × Rotiblock (Carl Roth) diluted in IP wash buffer (150 mM KCL, 10 mM Tris pH 7.5, 0.1 mM EDTA and 10% glycerol) and simultaneously incubated with 5 µg antibody for 30 min with gentle rotation at 4 °C: either BRG1-biotin (ab200911, Abcam), DDIT3-biotin (NB600-1335B, Novus Biologicals), FLI1-biotin (246159-biotin, US biologicals), or Normal mouse IgG-biotin antibody (sc-2762, Santa Cruz Biotechnology; negative control). The beads were then washed three times in IP wash buffer to remove unbound antibody and incubated with 50 µg nuclear extract (diluted to 250 µl with IP wash buffer supplemented with 1 × protease inhibitor) overnight with gentle rotation at 4 °C. The beads were immobilized on a magnet and the nonbound fraction was collected and mixed with 4 × NuPAGE LDS sample buffer (Thermo Fisher Scientific). Following two washes with IP wash buffer for 5 min on gentle rotation at 4 °C and two times 5 min incubation with wash buffer suitable for mass spectrometry (ultrapure water with 10 mM triethylammonium bicarbonate, Thermo Fisher Scientific), the beads were resuspended a third time in a smaller volume to concentrate the beads and transferred to a new tube to reduce unspecific protein binding from the tube surface. Co-immunoprecipitated proteins were eluted twice at 90 °C, 500 rpm for 10 min in 25 µl 2 × NuPAGE LDS sample buffer containing 10% NuPAGE sample reducing agent (Thermo Fisher Scientific), and pooled. All samples were stored at −20 °C. The IP yield was checked with western blot as previously described [7] before samples were analyzed with QMS.

### Quantitative mass spectrometry

Samples were preprocessed, trypsin-digested, labeled with tandem mass tag labels (TMTpro-16plex, Thermo Fischer Scientific), pooled, prefractionated, and analyzed with liquid chromatography–tandem mass spectrometry (LC–MS/MS) at the Proteomics core facility at University of Gothenburg; for details see Supplementary Methods, Additional file 1. Fractions were analyzed with an orbitrap Lumos Tribrid spectrometer connected to an Easy-nLC1200 nanoflow liquid chromatography system (both Thermo Fisher Scientific). Proteins were identified and quantified using Proteome Discoverer (v.2.4, Thermo Fisher Scientific).

The QMS analysis generated a list of peptides, the proteins they matched to using Swissprot Homo Sapiens database (3832) and the relative abundance of TMT-reporter ions for each IP sample. Only unique peptides were used for protein quantification. Data processing was performed in R (v.4.0.3) and Excel. Proteins not passing the quantification cutoff (see Supplementary Methods, Additional file 1 for details) as well as proteins only detected in IgG controls were excluded (698 and 3, respectively). Missing values were replaced with the lowest detected value. Data from each specific IP (either BRG1 or DDIT3) was then compared with its IgG control, generating mean abundance and fold change for each protein. At this point, the FLI1 IP was excluded from downstream analysis since the yield was too low; the mean enrichment of SWI/SNF core components compared with the negative control IgG in two of the replicates were below 1.5. For the remaining samples, proteins < 1.5 fold change compared with IgG control were removed from further analysis, leaving 3058, 3064, and 3064 protein interactors for DDIT3 IP, BRG1 IP in MLS and BRG1 IP in EWS, respectively. In the comparisons between BRG1 IP in MLS and BRG1 IP in EWS cells and between DDIT3 IP and BRG1 IP in MLS, only proteins remaining in both IP datasets were used, i.e., > 1.5 fold change compared with IgG control for both IPs, leaving 3046 and 3045 proteins (including BRG1), respectively. To analyze the SWI/SNF composition and enrichment of interaction partners, protein abundances were normalized against the amount of BRG1 in each IP replicate. Data were then analyzed in R based on fold change (foldchange function, gtools), *p*-value from unpaired Student’s *t*-test assuming nonequal variance (t.test function), and false-discovery rate based on Benjamini–Hochberg correction (p.adjust function, method BH). A 20% enrichment in either IP in the comparisons with *p*-value < 0.05 was considered significant. The QMS raw data including peptide abundances for all proteins retained in the analysis are shown in Supplementary Table 1, Additional file 2.

For downstream interaction partner analysis, we extracted previously reported BRG1-interacting partners (299 proteins) from NCBI/BioGRID (30) and histone variants (59 histones) in nextprot database [31], and utilized the Lambert et al. database [32] of human transcription factors (1639 proteins). Functional enrichment analysis of interacting proteins was performed in R using gene set collections including GO Biological processes, Genetic and Chemical perturbations, and Reactome from the molecular signature database (v 7.4) [33], as described previously [25]. Network analysis was performed in Cytoscape [34] using default settings.

### ATAC and RNA sequencing

The chromatin accessibility of MLS 1765–92 and EWS TC-71 cell lines (*n* = 3) was assessed with ATAC-seq [35]. Gene expression of MLS (402–91, 1765–92, and 2645–94) and EWS (TC-71 and 6647) cell lines (*n* = 4) was assessed with RNA-seq using the Smart-seq2 protocol [36] with minor modifications. Differential accessibility and expression analysis was performed using DESeq2. Single-cell RNA-seq was performed on 5000 MLS 402–91 cells using the PIPseq T2 protocol (Fluent BioSciences). Detailed protocols are described in Supplementary Methods, Additional file 1. ATAC-seq primers are shown in Supplementary Table 2, Additional file 3.

## Results

### Development of a quantification strategy to determine SWI/SNF complex composition and interactome in FET sarcoma

To determine the composition of SWI/SNF complexes and their interactomes in FET sarcoma, we developed a strategy consisting of IP from nuclear extracts followed by QMS analysis using TMT labels, a global approach for relative protein quantification. We pulled down either all SWI/SNF complexes using BRG1 as a target (BRG1 IP) in MLS 402–91 and EWS TC-71 cells or FET-FOP-bound SWI/SNF complexes using DDIT3 (DDIT3 IP) in MLS 402–91 cells (Supplementary Fig. 1A, Additional file 1). Note that the wild-type DDIT3 protein is not expressed during standard growth conditions. To verify the yield of the immunoprecipitation and identify specific interacting proteins, we compared the BRG1 and DDIT3 IP with the respective IgG control (Fig. 1B,C, Supplementary Fig. 1B,C, Additional file 1 and Supplementary Table 3, Additional file 4). We obtained low background signals in the immunoprecipitation as well as reproducible IP replicates. The BRG1 IP and DDIT3 IP were efficient, with > 15-fold enrichment of the target protein. Furthermore, the majority of co-immunoprecipitated proteins were enriched > 4 times compared with the negative IgG control. Most interactors were shared between the different IPs indicating a similar interactome for the fusion protein and SWI/SNF in these cells (Supplementary Fig. 1D, Additional file 1). To validate our approach, we confirmed that known FUS::DDIT3 interaction partners were identified and characterized them on the basis of protein and gene set databases, identifying expected terms such as chromosome organization and mRNA processing (Supplementary Fig. 1E–I, Additional file 1 and Supplementary Table 4, Additional file 5).

We aimed for a sensitive analysis, retaining all potentially important interaction partners. To quantify the variation in SWI/SNF composition and interactions, each IP sample was first normalized against the amount of BRG1 and then two comparisons were performed: (1) BRG1 IP in MLS versus BRG1 IP in EWS and (2) DDIT3 IP versus BRG1 IP in MLS (Fig. 1D and Supplementary Fig. 1J, Additional file 1). We identified 1299 significantly enriched proteins for BRG1 IP in MLS cells and 571 in EWS cells (Fig. 1D). In the second comparison, 2854 proteins were significantly enriched in the DDIT3 IP and 10 in the BRG1 IP in MLS. Not all of these proteins are believed to be direct interaction partners. The high number of detected interactors can be explained by several factors: (1) The applied QMS method is sensitive. Hence, many more proteins are identified compared to conventional MS analysis. (2) SWI/SNF components and FET-FOPs are prone to form interactions by, e.g., IDR regions and phase separation [2, 37]. Hence, many interactions are expected to occur in the nuclear extract during IP when proteins are in close proximity to each other. Interactions are enhanced by the fact that SWI/SNF complexes are abundant and contain numerous proteins with distinct interaction domains. (3) Nuclear isolation is not 100% efficient. Hence, abundant proteins from other cellular compartments remain in the lysate and may form nonnative interactions. Our experimental approach using IP-QMS allowed sensitive protein identification as well as quantitative information of relevant interaction partners. We focused most of our analyses on the 362 proteins defined as SWI/SNF components, SWI/SNF interaction partners, transcription factors, and histones. However, the total IP-QMS data set constitutes a valuable resource for future SWI/SNF and FET-FOP research.

### Distinct SWI/SNF complex compositions in MLS and EWS

**Fig. 2:**
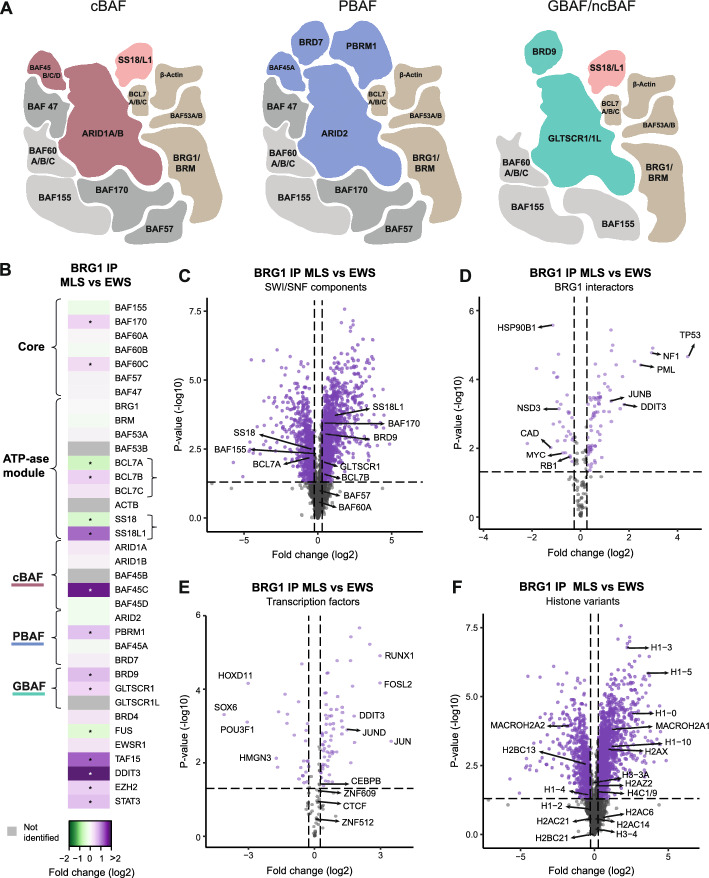
Composition of SWI/SNF complexes and interactions in MLS and EWS cells. **A** Schematic illustration of the three main SWI/SNF subtypes cBAF, PBAF, and GBAF/ncBAF. Core components shared between all subtypes (light gray) and only cBAF and PBAF (dark gray) are assembled before subtype-specification by addition of cBAF-specific components (maroon), PBAF-specific components (blue), and GBAF-specific components (green). The ATP-ase module (gold and peach) with the catalytic subunit BRG1 (or BRM) is added last. SWI/SNF paralogs, subunits that exist in different variants and are assembled into the complex in a mutually exclusive manner, such as BAF60A, BAF60B, and BAF60C, are indicated within the same component. **B** Heatmap showing the enrichment of SWI/SNF components and interaction partners in MLS 402–91 versus EWS TC-71 using BRG1 IP-QMS data. Certain paralogs that vary between MLS and EWS cells are indicated by curly brackets. *Fold change > 1.2 and *p*-value < 0.05, unpaired Student’s *t*-test. **C** Volcano plot visualizing the enrichment of interacting proteins in MLS versus EWS using BRG1 IP-QMS data, highlighting SWI/SNF components (*n* = 3044). **D** Volcano plot visualizing the enrichment of known BRG1 interactors in MLS versus EWS using BRG1 IP-QMS data (*n* = 163). **E** Volcano plot visualizing the enrichment of transcription factors in MLS versus EWS using BRG1 IP-QMS data (*n* = 144). **F** Volcano plot visualizing the enrichment of histone proteins in MLS versus EWS using BRG1 IP-QMS data (*n* = 3044). (**C**–**F**) Dashed lines indicate applied cutoff values for fold change: 1.2 and *p*-value: 0.05. Each dot indicates a protein, either non-significantly enriched (gray) or significantly enriched (purple)

Several variants of SWI/SNF complexes, including the three most common subtypes (Fig. 2A), can exist in the same cell and at the same time. In FET sarcoma cells, the SWI/SNF compositions remain largely undefined. Therefore, we analyzed the SWI/SNF components in MLS and EWS cells using the BRG1 IP-QMS data (Supplementary Table 5, Additional file 6). Four SWI/SNF components were not detected in any of the IPs: GLTSCR1L, BAF45B, BAF53B, and ACTB, likely owing to low expression levels for the first three while no unique peptides were identified for ACTB. Importantly, we detected no loss of any additional SWI/SNF component in either MLS or EWS. However, we observed significant differences in 10 out of 29 SWI/SNF components, mainly in subunit paralogs where BCL7B and SS18L1 were enriched in MLS cells, while BCL7A and SS18 were enriched in EWS (Fig. 2B, C). Furthermore, MLS cells contained more GBAF complexes than EWS; GLTSCR1 and BRD9 were enriched on average 1.4 times in MLS cells compared with EWS while the other subtype-specific components showed no consistent overall trend (Fig. 2B). However, the cBAF-specific BAF45C paralog was highly enriched and the PBAF-specific component PBRM1 was slightly enriched in MLS cells. In summary, these results point to distinct SWI/SNF complex compositions as well as an altered SWI/SNF-subtype distribution in different types of FET sarcoma cells.

### Distinct SWI/SNF interaction partners in MLS and EWS

To assess differences of SWI/SNF complexes in FET sarcoma cells, we analyzed all SWI/SNF interactions by comparing the BRG1 IPs in MLS and EWS cells (Supplementary Table 5, Additional file 6). Among known FUS::DDIT3 and SWI/SNF interaction partners, normal FUS was less associated with SWI/SNF complexes in MLS compared with EWS, while TAF15, STAT3, and EZH2 were enriched (Fig. 2B). Besides EZH2, two other PRC2 components, EED and SUZ12, were also enriched in MLS (Supplementary Table 5, Additional file 6). In addition, we identified 163 out of 299 (55%) previously reported BRG1 interaction partners in the FET sarcomas (Supplementary Table 6, Additional file 7). In total, 69 of the known BRG1 interactors displayed increased interaction with SWI/SNF in MLS cells (e.g. TP53, NF1, PML, and JUNB) and 31 in EWS cells (e.g. HSP90B1, NSD3, DNMT3A, CAD, MYC, and RB1) (Fig. 2D).

The SWI/SNF complex generally opens chromatin and regulates transcription through interaction with transcription factors. We identified 9% (145 out of 1639) of all predefined transcription factors [32] as interacting partners to SWI/SNF complexes in the two assessed FET sarcomas (Fig. 2E and Supplementary Table 7, Additional file 8). In MLS, 68 transcription factors displayed increased interaction with SWI/SNF. Among the top enriched were RUNX1, FOSL2, TEAD1, JUN, and JUND, predominantly known DDIT3 dimerization partners. These, as well as STAT3 and GATA2, were major nodes in a transcription factor protein network (Supplementary Fig. 2A, Additional file 1). Surprisingly, CEBPβ, a well-known interaction partner of FUS::DDIT3 [38], was not significantly enriched in SWI/SNF complexes in MLS cells compared with EWS. Furthermore, 26 transcription factors were enriched in EWS, including HOXD11, SOX6, POU3F1, FOXP1, and HMGN3, as well as YY1, USF2, and KDM5B, which were also major nodes in the transcription factor network (Fig. 2E and Supplementary Fig. 2A, Additional file 1). Enriched MLS transcription factor families included the basic leucine zipper (bZIP) family that DDIT3 is part of, as well as TEA, SAND, AT hook, and STAT families, while EWS was enriched for HMG/SOX and homeodomains families (Supplementary Table 7, Additional file 8). These data suggest that the SWI/SNF complex utilizes distinct sets of transcription factors in the two investigated FET sarcomas. However, 50 transcription factors displayed similar interactions with SWI/SNF in the two cancer types, such as several zinc-finger transcription factors (ZNF512, ZNF598, ZNF609, ZNF687, and ZNF709), CTCF, ELF2, and YBX1, indicating shared gene regulation networks (Fig. 2E and Supplementary Table 7, Additional file 8). Shared transcription factors CTCF, SP1, and CEBPβ were all major nodes in the interaction network (Supplementary Fig. 2A, Additional file 1). There was a weak correlation between the gene expression level and the protein enrichment of transcription factors as well as SWI/SNF subunits in SWI/SNF complexes in MLS and EWS (Supplementary Fig. 2B, C, Additional file 1), indicating that protein interactions more than abundances were important for the enriched SWI/SNF interactions.

SWI/SNF complexes interact with, and remodel, nucleosomes. These interactions rely on both post-translational modifications on the histone tails and the final SWI/SNF complex–nucleosome architecture [39]. To determine the nucleosome composition bound to SWI/SNF complexes in FET sarcoma cells, we analyzed the occurrence of histone variants (Fig. 2F, Supplementary Fig. 2D, Additional file 1 and Supplementary Table 8, Additional file 9). Among the 59 known histone variants, 18 were detected in the BRG1 IP data. Certain linker histone H1 variants (H1-0, H1-3, H1-5, and H1-10) were enriched in MLS cells, while others (H1-2 and H1-4) were enriched in EWS cells. Histone H1 is implicated as a driver in certain cancers with H1 variants showing diverse expression patterns in different tumor types [40]. There were also differences in the H2A variants that control the access to DNA [41], where H2AX, known to be involved in DNA damage response, was enriched in MLS cells. In addition, two other variants of histone H2A called MACROH2A1 and MACROH2A2 were enriched in MLS and EWS, respectively. Both are reported to repress transcription, inhibit transcription factor binding and suppress the remodeling activity of SWI/SNF complexes [42, 43]. In summary, SWI/SNF complexes in MLS and EWS have distinct interaction partners, notably transcription factors belonging to different families. The diverse SWI/SNF interactions is likely one way that different FET sarcomas enable unique gene expression patterns.

### Distinct chromatin accessibility and gene expression patterns in MLS and EWS

**Fig. 3:**
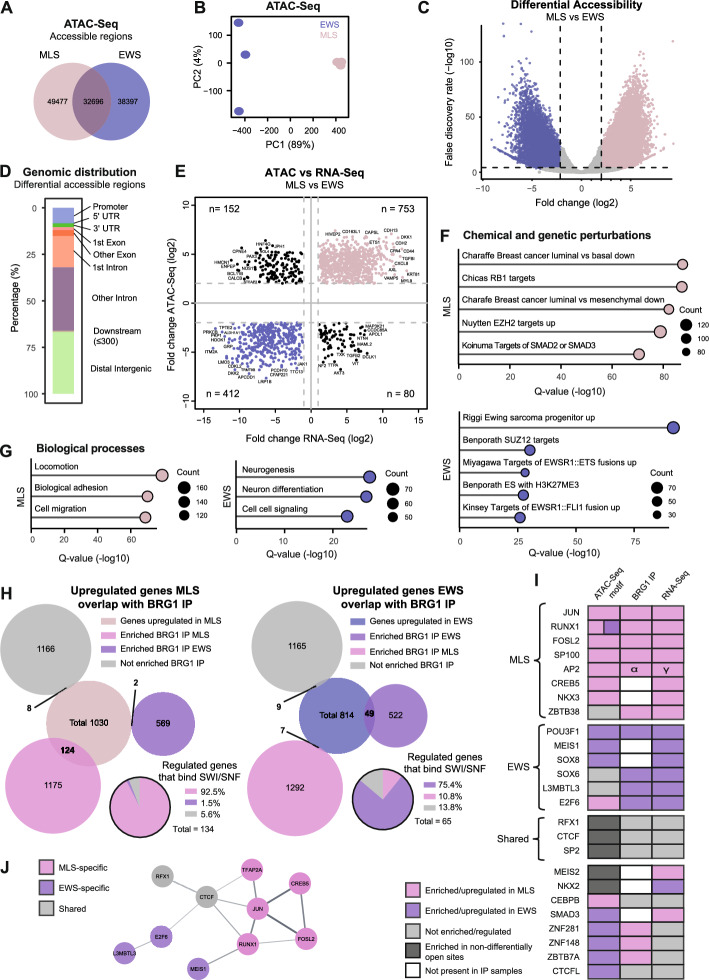
Differentially accessible regions and regulated genes in MLS and EWS cells. **A** Venn diagram of open chromatin regions for MLS 1765–92 and EWS TC-71 using ATAC-seq data. **B** Principal component analysis of MLS and EWS replicates based on ATAC-seq peaks (*n* = 3). **C** Volcano plot of differentially accessible chromatin regions in MLS versus EWS based on ATAC-seq data (*n* = 125,938). Each dot indicates a chromatin region. Significantly differentially open regions in MLS (light pink) and EWS (blue) are shown, with fold change > 4 and false discovery rate < 0.001. **D** Genomic annotation of differentially accessible chromatin regions in MLS and EWS based on ATAC-seq data. **E** Correlation between genes with differentially open chromatin regions (fold change > 4) and relative gene expression (fold change > 2) for MLS versus EWS using ATAC-seq and RNA-seq data. The Spearman correlation coefficient was 0.58 with *p* < 0.001. **F**, **G** Enrichment of gene sets from (**F**) chemical and genetic perturbations and (**G**) biological processes using upregulated genes with differentially open chromatin regions in MLS or EWS. Top five or three gene sets based on *q*-value are shown. Gene count is indicated by dot size. **H** Overlap between upregulated genes and BRG1 IP-enriched proteins in MLS and EWS, respectively. Percentage of enriched proteins in each BRG1 IP compared with the regulated genes that bind to SWI/SNF are shown in the pie charts. **I** Identification of MLS-specific, EWS-specific and shared transcription factors using HOMER de novo transcription factor motifs from ATAC-seq, SWI/SNF-binding transcription factors from BRG1 IP, and regulated transcription factors from RNA-seq. For each dataset, transcription factors can be upregulated/enriched in MLS (pink), EWS (purple) or not enriched (gray). ATAC-seq TF motifs have a fourth option of being enriched in non-differentially open sites. Transcription factors identified in at least two of the datasets are displayed. **J** Protein interaction network of MLS-specific, EWS-specific and shared transcription factors, created in Cytoscape

To discern differences and similarities in the epigenetic landscape between MLS and EWS, we performed ATAC-seq in MLS 1765–92 and EWS TC-71 cells (Fig. 3A, B and Supplementary Fig. 3A–D, Additional file 1). Around 30% of the open chromatin regions were shared between MLS 1765–92 and EWS TC-71 (Fig. 3A). Differential accessibility analysis identified 52,036 genomic regions differentially open between MLS and EWS cell lines, of which 61% were more open in MLS and 39% in EWS (Fig. 3C). These regions were mainly located to introns or distal intergenic regions and annotated to 13,411 genes (Fig. 3D). Interestingly, a smaller fraction of the differentially open regions was located in promotors compared with all open regions, suggesting that, close to the transcription start site, the regulation of chromatin accessibility was more similar between the two cell types (Fig. 3D and Supplementary Fig. 3D, Additional file 1). We then performed de novo Homer motif analysis to identify enriched transcription-factor binding sites in differentially open chromatin regions (Supplementary Fig. 3E, Additional file 1 and Supplementary Table 9, Additional file 10). For MLS, the top enriched motif was JUN/FOS, known dimerization partners of FUS::DDIT3, while the top enriched de novo binding sites in EWS was the fusion oncoprotein EWSR1::FLI1.

The gene expression of three MLS (402–91, 1765–92, and 2645–94) and two EWS (TC-71 and 6647) cell lines were analyzed using RNA-seq (Supplementary Fig. 3F, G, Additional file 1). Principal component analysis showed that MLS cell lines clustered differently from the EWS cell lines (Supplementary Fig. 3G, Additional file 1). We performed pairwise comparisons between all MLS and EWS cell lines and found 1030 significantly upregulated genes in MLS and 814 in EWS (Supplementary Fig. 3F, Additional file 1 and Supplementary Table 10, Additional file 11). Among the 1844 differentially expressed genes, 76% overlapped with genes with differentially open chromatin regions (Supplementary Fig. 3H, Additional file 1 and Supplementary Table 11, Additional file 12). Furthermore, the chromatin accessibility correlated positively with the mRNA expression for most genes (83%) (Fig. 3E). Functional enrichment analysis of gene sets with genes that have increased chromatin accessibility and increased mRNA expression in MLS identified gene sets connected to PRC2, RB1 and SMAD2/3 targets, mesenchymal breast cancer, cell migration, and biological adhesion, while in EWS, enriched gene sets included EWSR1::FLI1, PRC2 complex and H3K27me3 targets, as well as neurogenesis and cell signaling processes (Fig. 3F, G). In summary, the chromatin accessibility, especially at introns and distal regions, differed between FET sarcoma subtypes resulting in FET sarcoma-specific transcription associated with distinct biological functions.

### MLS and EWS-specific transcription factors and genes are enriched with SWI/SNF complexes

SWI/SNF complexes directly affect gene expression. However, it is possible that the downstream translated proteins of regulated genes interact with SWI/SNF complexes and affect their function. To evaluate this, we compared the regulated genes between MLS and EWS cells with proteins interacting with the SWI/SNF complex (Fig. 3H and Supplementary Table 12, Additional file 13). Out of the 1030 genes upregulated in MLS, 124 were also enriched in SWI/SNF complexes at protein level (12%), while only 10 of the upregulated genes were either not enriched or enriched in SWI/SNF complexes in EWS. The same trend was detected in EWS; 49 of the 814 genes upregulated in EWS were enriched in SWI/SNF complexes (6%), while 16 were either not enriched or enriched in SWI/SNF complexes in MLS. Thus, most SWI/SNF-interacting proteins regulated at gene level were enriched with the SWI/SNF complex in MLS (93%) and EWS (75%). Interestingly, functional enrichment analysis showed that the genes both upregulated at RNA level and enriched at protein level in BRG1 IP were significantly enriched in FET-FOP target genes (Supplementary Fig. 3I, Additional file 1). This suggests that the regulated genes may affect the function of the SWI/SNF complex and contribute to the oncogenic setting in both MLS and EWS.

To investigate the regulation mechanism in more detail, we identified key transcription factors in the FET sarcomas by comparing the differentially open binding motifs with the 144 transcription factors that interacted with the SWI/SNF complex and the 133 transcription factors that were significantly regulated between MLS and EWS (Fig. 3I). We identified eight MLS-specific transcription factors (JUN, RUNX1, FOSL2, SP100, AP2, CREB5, NKX3, and ZBTB38) and six EWS-specific transcription factors (POU3F1, MEIS1, SOX8, SOX6, L3MBTL3, and E2F6) enriched and regulated in the same direction in at least two of the three datasets (Fig. 3I and Supplementary Table 13, Additional file 14). We also identified three MLS- and EWS-shared transcription factors (RFX1, CTCF, and SP2) that were enriched in the shared open chromatin regions, while eight transcription factors displayed varying patterns (Fig. 3I). Several of the FET-sarcoma-specific and shared transcription factors formed a protein interaction network, with MLS-specific JUN, RUNX1, FOSL2, and CREB5 as center nodes (Fig. 3J). All of these except RUNX1 belong to the bZIP transcription factor family. The FET-sarcoma-specific transcription factors are involved in regulation of several cellular processes in development and disease (Supplementary Table 13, Additional file 14). Together these data imply that the identified transcription factors may guide the SWI/SNF complexes to open transcription factor-binding sites in a FET-sarcoma-type-specific manner.

### FUS::DDIT3-bound SWI/SNF complexes are enriched in PBAF and GBAF components

**Fig. 4:**
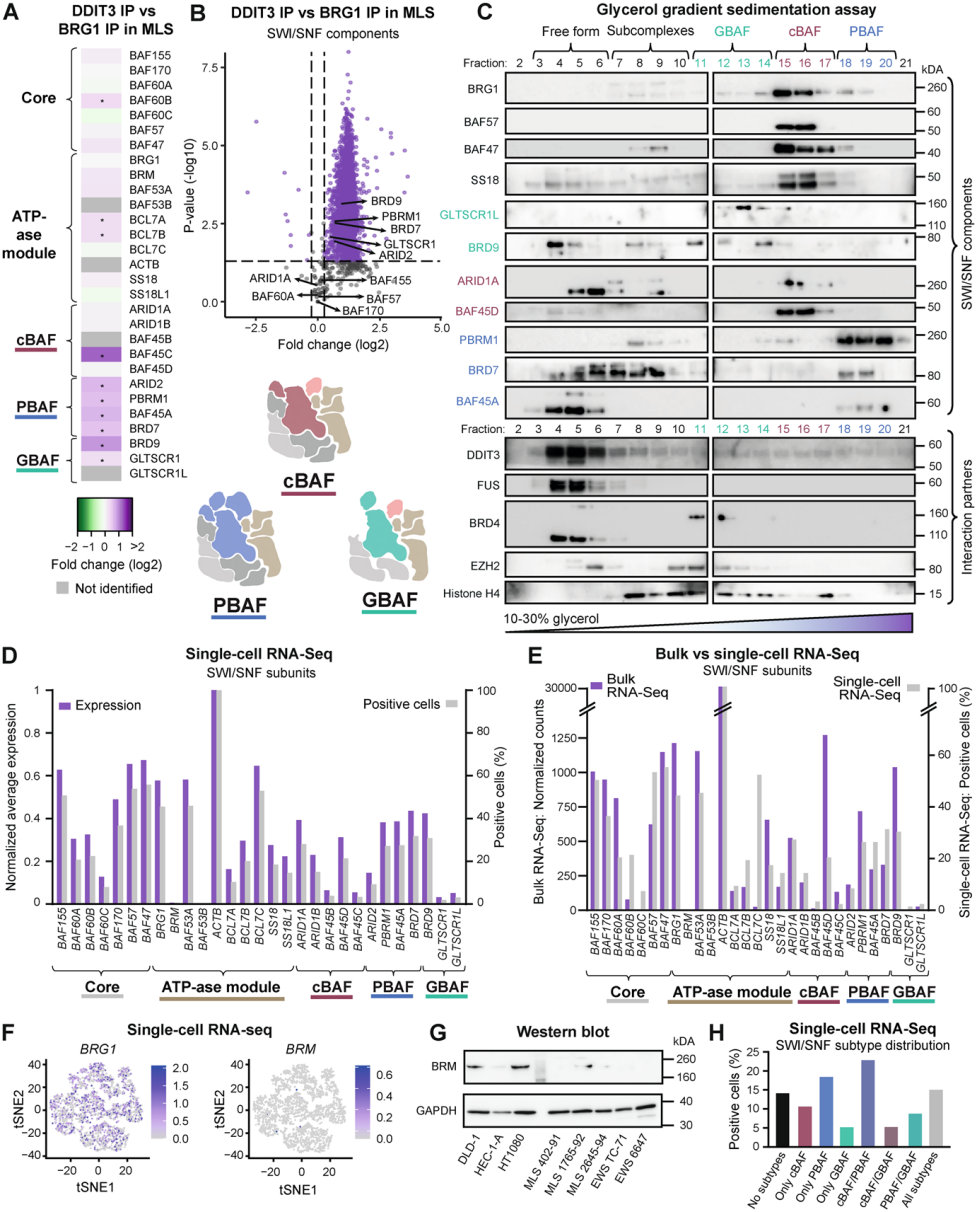
Composition of FUS::DDIT3-bound SWI/SNF complexes. **A** Heatmap showing the enrichment of SWI/SNF components in FUS::DDIT3-bound SWI/SNF complexes in MLS 402–91 cells using DDIT3 IP and BRG1 IP-QMS data. *Fold change > 1.2 and *p*-value < 0.05, unpaired Student’s *t*-test. **B** Volcano plot visualizing the enrichment of interacting proteins in FUS::DDIT3-bound SWI/SNF complexes in MLS using DDIT3 IP and BRG1 IP-QMS data, highlighting SWI/SNF components (*n* = 3045). Dashed lines indicate applied cutoff values for fold change: 1.2 and *p*-value: 0.05. Each dot indicates a protein, either non-significantly enriched (gray) or significantly enriched (purple). **C** Western blot analysis of fractions 2–21 from glycerol gradient sedimentation assays of nuclear extracts of MLS 402–91 visualizing size-separated SWI/SNF complexes in free form, subcomplexes, and the three subtypes GBAF, cBAF, and PBAF. Antibodies against core and subtype-specific SWI/SNF components and selected interactions partners FUS::DDIT3 (DDIT3 antibody), normal FUS, BRD4, EZH2, and Histone H4 are shown. **D** Average gene expression and percentage of positive cells for SWI/SNF components based on single-cell RNA-seq data in MLS 402–91 cells (*n* = 2923). Gene expression levels were normalized against *ACTB* for visualization purposes. **E** Average gene expression using bulk RNA-seq (*n* = 4) and percentage of positive cells using single-cell RNA-seq (*n* = 2923) for SWI/SNF components in MLS 402–91 cells. **F**. *t*-Distributed stochastic neighbor embedding (*t*-SNE) plots based on single-cell RNA-seq data on the whole transcriptome in MLS 402–91 cells (*n* = 2923 cells), where the specific expression of *BRG1* and *BRM* is shown. **G** Western blot analysis of BRM in whole-cell extracts of MLS 402–91, 1765–92, and 2645–94, and EWS TC-71 and 6647 cells compared with control cancer cell lines DLD-1, HEC-1-A, and HT1080, with GAPDH as loading control. **H**. Distribution of the three main SWI/SNF subtypes cBAF, PBAF, and GBAF in MLS 402–91 using single-cell RNA-seq data, based on cells positive for at least one core SWI/SNF component (*n* = 2893). Complete western blots are displayed in Supplementary Fig. 5A–C, Additional file 1

To determine the effects of FET oncoproteins on SWI/SNF complexes and their potential binding preferences, we compared the composition of FET-FOP-bound SWI/SNF (DDIT3 IP) with all SWI/SNF complexes (BRG1 IP) in MLS cells (Supplementary Table 14, Additional file 15). There was five times more FUS::DDIT3 per SWI/SNF complex in DDIT3 IP compared with BRG1 IP. FET-FOP-bound SWI/SNF complexes contained both core and subtype-specific components from all three major SWI/SNF subtypes cBAF, PBAF, and GBAF (Figs. 2A and 4A, B). The presence of FUS::DDIT3 in the complex caused no loss of any SWI/SNF component nor disrupted or changed the core composition (Fig. 4A, B). We observed significant differences in 10 out of 29 SWI/SNF components; some subunit paralogs were slightly enriched, such as BAF60B, BCL7A, and BCL7B. Furthermore, FUS::DDIT3-bound complexes were significantly enriched in PBAF and GBAF subtype-specific components with a mean enrichment of 1.6, suggesting that FUS::DDIT3 interacts more with fully formed PBAF and GBAF complexes (Fig. 4A, B and Supplementary Table 14, Additional file 15). To evaluate this further, we performed sedimentation assays, where SWI/SNF subtypes are separated based on size in a glycerol gradient (Fig. 4C). Western blot analysis showed detection of cBAF, PBAF and GBAF-specific components in expected fractions. Analysis of the core component BRG1, which is found in all subtypes, revealed that cBAF was the most abundant SWI/SNF subtype in MLS cells. Intriguingly, BAF57 and BAF47 were not only absent from GBAF complexes as expected, but they were barely present in PBAF complexes. Furthermore, we observed more free-form SWI/SNF subunits than reported [19, 44], for components such as SS18, BRD9, ARID1A, and BRD7. This analysis confirmed that FUS::DDIT3 interacts with all SWI/SNF subtypes. Although many of the transient protein interactions may have been released during the harsh ultracentrifugation, FUS::DDIT3, but not FUS, was present in complexes of all sizes.

To account for cell heterogeneity in SWI/SNF complex composition, we performed single-cell RNA-seq of MLS 402–91 cells focusing on the expression of SWI/SNF genes (Supplementary Table 15, Additional file 16). The percentage of positive cells for each SWI/SNF component correlated to their average expression at both single-cell and bulk level for most genes (Fig. 4D, E). *ACTB* was detected in all single cells, while the detection of SWI/SNF core components *BAF155*, *BAF170*, *BAF57*, *BAF47*, and *BRG1* ranged from 36% to 56% (Fig. 4D, F and Supplementary Fig. 4A, Additional file 1). Some subtype-specific components were identified in up to 30% of cells, whereas *BAF45B/C* and *GLTSCR1/1L* were only detected in few cells, potentially explaining why some of these components were not identified in the QMS analysis. Importantly, almost no MLS cells were positive for *BRM*, the mutually exclusive catalytic subunit to BRG1, indicating that very few complexes are missed when BRG1 is used as a target during immunoprecipitation. The low BRM expression in FET sarcoma cell lines was confirmed by western blot analysis (Fig. 4G). The low expression of *BAF45B/C*, *BAF53B*, *BRM*, and *GLTSCR1/1L* was verified in the bulk RNA-seq data (Fig. 4E). In the single-cell analysis, expression of around 4–10 SWI/SNF components were detected in most cells (77%) in varying distributions (Supplementary Fig. 4B, Additional file 1). Expression of cBAF, PBAF, and GBAF was detected in 54%, 65%, and 34% of the cells, respectively (Fig. 4H). In addition, most cells were positive for one or two subtypes (35% or 37%, respectively), while 15% contained all subtypes and 14% had no detected subtype. Note that the indicated percentages and number of genes are lower estimates, since the expression of most genes at single cell level is below the limit of detection in a given cell, as a consequence of transcriptional heterogeneity [45]. We observed no apparent clustering of cells based on their expression of SWI/SNF components in relation to the entire transcriptome (Fig. 4F and Supplementary Fig. 4A, Additional file 1). To determine whether SWI/SNF genes were co-expressed or expressed in a mutually exclusive manner, we compared the observed frequency of co-expressed genes with the likelihood that they are expressed in the same cells by chance (Supplementary Fig. 4C, Additional file 1 and Supplementary Table 16, Additional file 17). We observed no or weak dependencies for all gene pairs and conclude that there was no mutual exclusivity in expression of SWI/SNF genes, as have been reported for SWI/SNF subunit assembly. In summary, while FUS::DDIT3 binding caused no extreme effects on the SWI/SNF composition, several paralogs as well as PBAF and GBAF subtype-specific components were significantly enriched in FUS::DDIT3-bound SWI/SNF complexes, at levels that may impact the SWI/SNF function in FET sarcoma cells.

### FUS::DDIT3 increases recruitment of known interaction partners and specific transcription factors

**Fig. 5:**
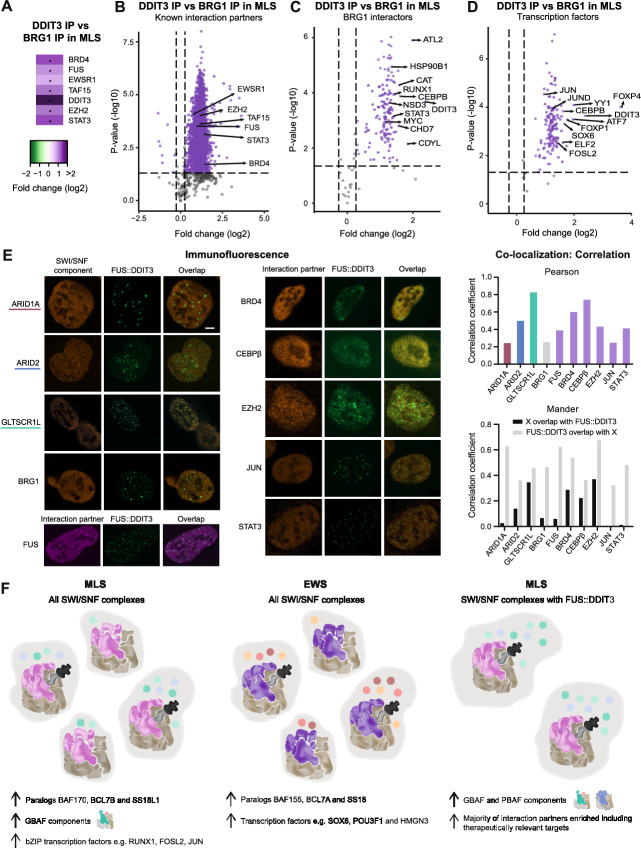
Enrichment of interaction partners in FET-FOP-bound SWI/SNF complexes. **A** Heatmap showing the enrichment of known FUS::DDIT3 interaction partners in FUS::DDIT3-bound SWI/SNF complexes in MLS 402–91 cells using DDIT3 IP and BRG1 IP-QMS data. *Fold change > 1.2 and *p*-value < 0.05, unpaired Student’s *t*-test. **B** Volcano plot visualizing the enrichment of known FUS::DDIT3 interacting partners in FUS::DDIT3-bound SWI/SNF complexes in MLS using DDIT3 IP and BRG1 IP-QMS data (*n* = 3045). **C** Volcano plot visualizing the enrichment of known BRG1 interactors in FUS::DDIT3-bound SWI/SNF complexes in MLS using DDIT3 IP and BRG1 IP-QMS data (*n* = 163). **D** Volcano plot visualizing the enrichment of transcription factors in FUS::DDIT3-bound SWI/SNF complexes in MLS using DDIT3 IP and BRG1 IP-QMS data (*n* = 145). In **B**–**D**, dashed lines indicate applied cutoff values for fold change: 1.2 and *p*-value: 0.05. Each dot indicates a protein, either non-significantly enriched (gray) or significantly enriched (purple). **E**. Immunofluorescence staining of HT1080 cells with transient expression of FUS::DDIT3-EGFP (green) forming small nuclear puncta. Staining with the core- and subtype-specific SWI/SNF components BRG1, ARID1A, ARID2, and GLTSCR1L, and interacting partners normal FUS, BRD4, CEBPβ, EZH2, JUN, and STAT3 (orange or purple), and their co-localization with FUS::DDIT3 (overlap) are displayed. Representative images are shown. Scale bar is 5 µm. Pearson’s and Mander’s split correlation coefficients for co-localization of FUS::DDIT3 and indicated proteins based on immunofluorescence staining of HT1080 cells with transient expression of FUS::DDIT3-EGFP. For Mander’s split coefficients, black bars correspond to the overlap between each protein with FUS::DDIT3 and light-gray bars correspond to the fraction of FUS::DDIT3 that overlap with each protein. **F** Schematic visualization of distinct SWI/SNF compositions and interaction partners in MLS and EWS as well as in FUS::DDIT3-bound SWI/SNF complexes in MLS, based on IP-QMS data

To further determine the effects of FET-FOP binding to SWI/SNF, we evaluated the impact of FUS::DDIT3 binding on known interaction partners by comparing the DDIT3 IP with BRG1 IP in MLS. Most known FUS::DDIT3 and SWI/SNF interaction partners, such as normal FET proteins (FUS, EWSR1, and TAF15), BRD4, EZH2, and STAT3, were enriched in FET-FOP-bound SWI/SNF complexes (Fig. 5A, B). The glycerol gradient sedimentation assay revealed that the 160 kDa isoform of BRD4 and the PRC2-component EZH2 primarily sedimented with GBAF complexes, while histone H4 sedimented with both subcomplexes and fully formed SWI/SNF subtypes (Fig. 4C). The co-immunoprecipitated proteins in FUS::DDIT3-containing complexes were on average 2.3 times enriched compared with all SWI/SNF complexes (Supplementary Table 14, Additional file 15). Except for the core SWI/SNF components, the majority (88%) of the known BRG1 interactors were enriched in FUS::DDIT3-bound complexes, such as ATL2, CAT, RUNX1, CEBPβ, CHD7, and CDYL (Fig. 5C and Supplementary Table 6, Additional file 7). Almost all (97%) SWI/SNF-interacting transcription factors were also enriched in the DDIT3 IP, e.g., YY1, FOXP4, FOXP1, ATF7, DOX6, and ELF2 (Fig. 5D and Supplementary Table 7, Additional file 8). The top enriched transcription factors mainly belonged to the bZIP and forkhead transcription factor families. To confirm co-localization of distinct interacting partners, we performed immunofluorescence experiments in a model system, staining cells after transient FUS::DDIT3-EGFP transfection. Endogenous FUS::DDIT3 is known to form small nuclear puncta dispersed over the whole nucleus, while overexpressed FET-FOPs form fewer, but larger, nuclear puncta with liquid-like characteristics [46]. We evaluated the co-localization between FUS::DDIT3-EGFP localized to relatively small, endogenous-like nuclear puncta with core- and subtype-specific SWI/SNF components BRG1, ARID1A, ARID2, and GLTSCR1L as well as interacting partners FUS, BRD4, CEBPβ, EZH2, JUN, and STAT3 (Fig. 5E). All stained proteins displayed a diffuse staining pattern. We observed co-localization with FUS::DDIT3 to various degrees, ranging from 0.37 to 0.63 (Mander’s split coefficient) for SWI/SNF components and from 0.33 to 0.68 for interaction partners. The abundance of enriched proteins suggests that in addition to the C-terminal dimer-forming DDIT3, the N-terminal FET partner, partly via its IDR, contributes with extensive binding properties. In summary, most interaction partners, including known interactors and bZIP transcription factors, were enriched in FUS::DDIT3-bound SWI/SNF complexes.

## Discussion

FET fusion oncoproteins are the main oncogenic drivers in around 20 types of sarcoma and leukemia, causing aberrant gene regulation via interactions with the SWI/SNF chromatin remodeling complex [7, 8, 25]. To delineate the specific role of FET oncoproteins and determine the SWI/SNF complex composition and interactome in FET sarcoma, we develop a quantitative strategy using IP-QMS. We identify distinct SWI/SNF complex compositions and interaction partners in MLS and EWS (Fig. 5F), resulting in different chromatin landscapes and gene expression patterns. Furthermore, we discover that FUS::DDIT3-bound SWI/SNF complexes are enriched in PBAF and GBAF components and that they have a larger interactome compared with all SWI/SNF complexes (Fig. 5F).

Our data reveal distinct SWI/SNF compositions in MLS and EWS cells, with alterations of certain SWI/SNF subunit paralogs and different subtype distribution, with increased amount of GBAF complexes in MLS. This potentially affects the SWI/SNF genome binding capacity since GBAF complexes mainly bind at CTCF regions and promoters, while cBAF localizes to enhancers, and PBAF binds to promoters and gene bodies [20–22, 44]. Furthermore, it is known that different SWI/SNF compositions, such as distinct subunit paralogs, are associated with cell-type-specific gene expression and cell identity, and that changes in the amount of available paralogs can lead to subunit switching, affecting the composition and function of the complex [47, 48]. We also show that the SWI/SNF complexes utilize distinct sets of transcription factors and interaction partners in FET sarcoma cells. We reveal that FET-sarcoma regulated genes including transcription factors have increased interactions with the SWI/SNF complex, potentially affecting its function and the oncogenic mechanism. Recently, the possible existence of a feedback loop between transcription factors and SWI/SNF was reported on the basis of the presence of SWI/SNF subunits in the promoters of transcription factors known to interact with the SWI/SNF complex [15], indicating an additional control mechanism that may be disrupted in FET sarcoma. Specifically, we find that bZIP transcription factors including known DDIT3 dimerization partners are enriched in MLS, while certain HMG/SOX transcription factors are enriched in EWS. Several of the identified MLS- and EWS-specific transcription factors are implicated in FET sarcoma oncogenesis; for example, increased gene expression of the EWS-specific transcription factors HOXD11, SOX6, MEIS1, and E2F6 were reported to be involved in EWS malignancy [49–53]. Furthermore, the MLS-specific transcription factor GATA2 was found to repress adipogenesis and block adipocyte differentiation [54], potentially a key mechanism in MLS development [55]. Interestingly, the MLS-specific factors JUN and FOSL2, members of the AP-1 complex and known DDIT3 dimerization partners [56], were found to bind the SWI/SNF complex and act as pioneering factors, targeting SWI/SNF complexes to inactive chromatin and inducing large epigenetic and chromatin reorganization [57]. It remains to be elucidated whether the different SWI/SNF compositions, subtypes or paralogs are responsible for the specific interaction partners identified in MLS and EWS. Our data suggest that the presence of FET-FOPs results in altered SWI/SNF composition, interactions, and function. This will most likely interplay with the distinct preexisting chromatin landscapes and gene expression programs of the precursor cells and contribute to the tumor development, in a distinct way for MLS and EWS. Indeed, oncogenesis of FET-FOPs has been attributed to certain contexts with a permitting epigenetic landscape where the cell of origin likely is a low-differentiated cell type, such as a tissue-specific progenitor or stem cell [6, 58–61].

Aberrant SWI/SNF complexes, formed after mutation in a SWI/SNF component or binding of a fusion oncoprotein, may be altered in a structural or functional way, i.e., either altered composition and structural integrity, or changed SWI/SNF activity and genomic targeting without affecting the structure [11, 62]. So far, no apparent structural effects of FET fusion binding have been reported, instead, FET-FOP-bound SWI/SNF complexes were shown to be highly stable up to 1 M salt [7, 25]. Here, we show that FUS::DDIT3 causes no disruption or change in the core composition or complete loss of any component. Instead, there is an enrichment of fully formed GBAF and PBAF complexes, as well as most interacting proteins, including the transcriptional co-activator BRD4 and the PRC2-component EZH2. SWI/SNF complexes have been reported to interact with, and oppose, both PRC1 and PRC2 complexes [7, 63–65]. The balance between SWI/SNF and PRC complexes controls DNA accessibility and expression of genes involved in lineage commitment and differentiation [66]. Therapeutic strategies against aberrant SWI/SNF complexes have emerged during the last decade [11]. These strategies rely on targeting vulnerabilities emerging in the cancer cells, such as EZH2 inhibition in BAF47-deficient cancer cells with decreased ability to oppose PRC2 function [11, 67–69], targeting synthetic lethal relationships between paralog SWI/SNF subunits, such as ARID1A/ARID1B [70] and BRG1/BRM [71, 72], or targeting specific SWI/SNF subtypes, such as GBAF in certain BAF47-deficient tumors [22, 44, 73]. FET sarcoma cells are sensitive to BRD4 inhibition, possibly by disrupting the genomic binding of BRD4, FET-FOPs, and SWI/SNF complexes [25]. It remains to be determined whether FET sarcomas share any other SWI/SNF-related therapeutic vulnerabilities.

The comparison of FET-FOP-bound SWI/SNF with all SWI/SNF complexes was performed with the assumption that most fusion protein bind to SWI/SNF complexes. Indeed, published studies indicated that around 80% of all FET-FOPs are bound to SWI/SNF [7, 25], which is supported by our IP-QMS data that show that FUS::DDIT3 share the majority of the interactome with BRG1. Our data reveal five times more FUS::DDIT3 per SWI/SNF complex in DDIT3 IP than BRG1 IP. However, this does not imply that 20% of SWI/SNF complexes are bound to a fusion protein, since several FUS::DDIT3 molecules may bind to each complex. SWI/SNF complexes are abundant and dispersed throughout the nucleus and the majority of complexes are likely not bound to FET fusion proteins at a given time point [8]. FET oncoproteins interact with SWI/SNF in a robust but transient manner that does not require DNA and may occur outside the chromatin environment [7, 25]. While several IP studies point to a high proportion of FET-FOP binding to SWI/SNF [7, 8, 25], the glycerol gradient sedimentation assay raises the question of how large proportion of FET-FOPs remain free. The experimental conditions differ between the IP and the glycerol gradient experiments, which may also explain the observed dissimilarity. This remains to be fully elucidated with future experiments. Our results indicate that FUS::DDIT3 mainly interacts with fully assembled SWI/SNF complexes. While it is known that only fully formed SWI/SNF complexes display chromatin remodeling activity, since the ATP-ase module is assembled last [13, 19], it remains to be determined whether SWI/SNF interactions are dependent on the formation of complete and activated complexes. In support of this, we find that most known SWI/SNF interactors are enriched in FUS::DDIT3-bound complexes, including transcription factors belonging to the bZIP family such as ATF7, CEBPβ, FOSL2, and JUN. Thus, our data suggest that FUS::DDIT3 acts together with fully functional complexes and not by inhibiting SWI/SNF assembly or cofactor recruitment.

The FET N-terminal domains of FET-FOPs contain prion-like IDR domains that interact extensively with other IDR proteins and possess phase separation potential, forming nuclear puncta referred to as biocondensates or hubs [2, 74]. The enrichment of interactors by FUS::DDIT3 suggests that FET-FOPs can form hubs with high concentration of oncoproteins and interaction partners, most probably at specific genomic sites, as observed for other IDR-containing transcription factors [75]. Recent data suggest that part of the oncogenic function of FET-FOPs occurs through IDR interactions and liquid–liquid phase separation [8, 37, 46, 75, 76]. For example, EWSR1::FLI1 enrichment by EWSR1 IDR–IDR interactions was required for SWI/SNF recruitment and transcriptional activation at GGAA microsatellites [8, 75]. Additionally, FET-FOPs including EWSR1::FLI1 and FUS::GAL4 formed biocondensates at specific loci, recruited RNA polymerase II and enhanced gene transcription [76]. Not only are the IDR interactions a probable explanation for FET-FOP and SWI/SNF interactions [37], but protein interactions mediated by the ARID1A IDR in cBAF were shown to be important for correct function of the chromatin remodeling complex [77]. Furthermore, the transactivation domain of transcription factors, which in many cases contain IDR domains, have been reported to be important for interactions with SWI/SNF components [13, 75, 78]. Although other types of protein interactions are relevant, IDR domains contribute with extensive protein interactions which, together with the C-terminal DNA-binding domain of FET-FOPs, are important for their oncogenic mechanism.

The current study has some limitations. All data were generated from experimental in vitro systems using a restricted number of cancer cell lines, which may deviate from clinical samples. The study is focused on proteomic analysis of SWI/SNF complex composition and interaction partners. Additional validation experiments using complementary methods would strengthen the conclusions. Our results suggest a potential impact of SWI/SNF components and interacting transcription factors on the downstream epigenetic landscape, findings that have not been experimentally validated. The function of distinct SWI/SNF complexes and interaction partners, such as the contribution of individual transcription factors, SWI/SNF subtypes, and components at specific genomic loci, remains to be determined.

## Conclusions

In this study, we develop a comprehensive IP-QMS approach to determine the impact of FET-FOPs on SWI/SNF composition and interactome to elucidate the role of aberrant SWI/SNF function in FET sarcoma. We define distinct differences in SWI/SNF composition, interaction partners, and epigenetic landscapes in MLS and EWS that may contribute to the tumor type specificity of FET-FOPs. We discover that FUS::DDIT3-bound complexes in MLS are enriched in PBAF and GBAF components, as well as therapeutically relevant interaction partners. Our data suggest that FET oncoproteins act together with fully assembled and functional SWI/SNF complexes and recruited interaction partners. Improved understanding of the molecular mechanisms of FET oncoproteins in tumorigenesis provides a basis for targeted interventions for the entire FET sarcoma family.

## Supplementary Information

Additional file 1. Supplementary information: Supplementary methods and figures.

Additional file 2. Supplementary Table 1.

Additional file 3. Supplementary Table 2.

Additional file 4. Supplementary Table 3.

Additional file 5. Supplementary Table 4.

Additional file 6. Supplementary Table 5.

Additional file 7. Supplementary Table 6.

Additional file 8. Supplementary Table 7.

Additional file 9. Supplementary Table 8.

Additional file 10. Supplementary Table 9.

Additional file 11. Supplementary Table 10.

Additional file 12. Supplementary Table 11.

Additional file 13. Supplementary Table 12.

Additional file 14. Supplementary Table 13.

Additional file 15. Supplementary Table 14.

Additional file 16. Supplementary Table 15.

Additional file 17. Supplementary Table 16.

## Data Availability

The quantitative mass spectrometry proteomics data have been deposited to the ProteomeXchange Consortium via PRIDE with the dataset identifier PXD047503. The ATAC-seq data was uploaded to NCBI’s Gene expression omnibus (accession number GSE235218). RNA-seq and single-cell RNA-seq data were uploaded to NCBI’s Gene expression omnibus (accession numbers GSE248354 and GSE248661). In addition, processed data utilized in this study are displayed in the Supplementary Tables, Additional file 2–17.

## Abbreviations

ATAC-seq: Assay for transposase-accessible chromatin sequencing
EDTA: ethylenediaminetetraacetic acid
EWS: Ewing sarcoma
FASP: Filter-aided sample preparation
FET: *FUS*, *EWSR1*, and *TAF15*
FET-FOP: FET fusion oncoprotein
IDR: Intrinsically disordered region
IP: Immunoprecipitation
LC-MS/MS: liquid chromatography- tandem mass spectrometry
MLS: Myxoid liposarcoma
PRC2: Polycomb repressive complex 2
PSM: Peptide-spectrum match
t-SNE: t-Distributed stochastic neighbor embedding
TMT: Tandem mass tag
QMS: Quantitative mass spectrometry
