# Supplementary material for: FET fusion oncoproteins enrich SWI/SNF complex subtypes and interaction partners
Source: Cell Mol Biol Lett. 2025 Sep 23;30:107. doi: 10.1186/s11658-025-00792-w (PMC12455829; doi:10.1186/s11658-025-00792-w)
Supplement: Supplementary file 1 — Additional file 1. Supplementary information: Supplementary methods and figures. [file 11658_2025_792_MOESM1_ESM.pdf]

FET fusion oncoproteins enrich SWI/SNF complex subtypes and interaction partners

Malin Lindén[1], Lisa Andersson[1], Heba Albatrok[1], Vilma Canfjorden[1], Emma Jonasson[1], Kajsa Grönqvist[1], Daniel Sjövall[2], Pekka Jaako[2], Rossella Crescitelli[3], Henrik Fagman[1], Pierre Åman[1], Anders Ståhlberg[1, 4, 5, 6*].

[1] Sahlgrenska Center for Cancer Research, Department of Laboratory Medicine, Institute of Biomedicine, Sahlgrenska Academy, University of Gothenburg, Gothenburg, Sweden
[2] Sahlgrenska Center for Cancer Research, Department of Microbiology and Immunology, Institute of Biomedicine, Sahlgrenska Academy, University of Gothenburg, Gothenburg, Sweden
[3] Sahlgrenska Center for Cancer Research, Department of Surgery, Institute of Clinical Sciences, Sahlgrenska Academy, University of Gothenburg, Gothenburg, Sweden
[4] Wallenberg Centre for Molecular and Translational Medicine, University of Gothenburg, Gothenburg, Sweden
[5] Region Västra Götaland, Sahlgrenska University Hospital, Department of Clinical Genetics and Genomics, Gothenburg, Sweden
[6] Science for Life Laboratory, Institute of Biomedicine, University of Gothenburg, Gothenburg, Sweden

Additional file 1. Supplementary information

Supplementary methods 2

Quantitative mass spectrometry (extended protocol) 2
ATAC sequencing (extended protocol) 3
ATAC sequencing data analysis 4
RNA sequencing (extended protocol) 4
RNA sequencing data analysis 5
Single-cell RNA sequencing and data analysis (extended protocol) 5
Glycerol gradient sedimentation assay 8
Whole-cell protein extraction 8
Western blot 8
Immunofluorescence 8

Supplementary Figures 10

Supplementary Figure 1. IP-QMS: Experimental design, quality control and validation 10
Supplementary Figure 2. Interactions and correlation of transcription factors in MLS and EWS. 12
Supplementary Figure 3. ATAC-Seq and RNA-Seq comparison of MLS and EWS cells. 13
Supplementary Figure 4. Single-cell RNA-Seq analysis. 15
Supplementary Figure 5. Complete western blot membranes. 17

Supplementary Table legends 19

Supplementary Table 1. QMS raw data 19
Supplementary Table 2. ATAC-Seq primers 19
Supplementary Table 3. Specific interactors IP vs IgG 19
Supplementary Table 4. Characterization and enrichment FUS::DDIT3 interactors 19
Supplementary Table 5. Proteins analyzed in comparison between BRG1 IP MLS and EWS (comparison 1) 19
Supplementary Table 6. BRG1 interaction partners 19

Supplementary Table 7. Transcription factors 19
Supplementary Table 8. Histone variants 19
Supplementary Table 9. Differentially open motifs 19
Supplementary Table 10. Differentially expressed genes between MLS and EWS 20
Supplementary Table 11. ATAC- and RNA-Seq data 20
Supplementary Table 12. Overlap of regulated genes and enriched proteins in BRG1 IP in MLS vs EWS 20
Supplementary Table 13. Enrichment, characterization and function of FET-sarcoma-specific transcription factors... 20
Supplementary Table 14. Proteins analyzed in comparison between DDIT3 IP and BRG1 IP MLS (comparison 2) 20
Supplementary Table 15. Single-cell RNA-Seq data 20
Supplementary Table 16. Single-cell SWI/SNF gene co-expression 20
References 20

Supplementary methods

Quantitative mass spectrometry (extended protocol)

Immunoprecipitated samples were analyzed with LC-MS/MS at the Proteomics core facility at University of Gothenburg. Immunoprecipitated samples were reduced with 100 mM DL-dithiothreitol (DTT) at 60°C for 30 min and then processed according to a modified version of the filter-aided sample preparation (FASP) method (1). Briefly, reduced samples were transferred onto Microcon-30kDa centrifugal filters (Merck), washed repeatedly with 50 mM triethylammonium bicarbonate (TEAB) and once with digestion buffer (0.5 % sodium deoxycholate (SDC), 50 mM triethylammonium bicarbonate, TEAB). The reduced cysteine side chains were alkylated with 10 mM methyl methanethiosulfonate (MMTS) in digestion buffer for 30 min at room temperature and the samples were then repeatedly washed with digestion buffer. Samples were digested with 0.3 µg trypsin (Pierce MS grade Trypsin, Thermo Fisher Scientific) at 37°C overnight followed by an additional portion of 0.3 µg trypsin and incubation for another 3 h. The peptides were collected by centrifugation and isobaric labeling was performed using Tandem Mass Tag reagents (TMT-16plex, Thermo Fischer Scientific) according to the manufacturer's instructions. The labelled samples were combined into one pooled sample, acetonitrile was evaporated using vacuum centrifugation and sodium deoxycholate (SDC) was removed by acidification with 10% trifluoroacetic acid (TFA). The sample was further purified using HiPPR Detergent Removal Spin Column (Thermo Fisher Scientific) and Pierce peptide desalting spin columns (Thermo Fisher Scientific), according to the manufacturer's instructions. The combined TMT-labeled sample was fractionated into 40 fractions by basic reversed-phase chromatography (bRP-LC) using a Dionex Ultimate 3000 UPLC system (Thermo Fischer Scientific) Peptide were separated using an XBridge BEH C18 column (3.5 µm, 3.0x150 mm, Waters Corporation) and a linear gradient from 3% to 40% acetonitrile (ACN) in 10 mM ammonium formate at pH 10.00 over 17 min followed by an increase to 90% acetonitrile over 5 min. The primary fractions were concatenated into 10 fractions, evaporated and reconstituted in 20 µl of 3% acetonitrile, 0.2% trifluoroacetic acid (TFA) for nLC-MS/MS analysis.

Samples were analyzed on an orbitrap Lumos Tribrid spectrometer connected to an Easy-nLC1200 nanoflow liquid chromatography system (both Thermo Fisher Scientific). Peptides were trapped on an Acclaim Pepmap 100 C18 trap column (100 µm x 2 cm, particle size 5 µm, Thermo Fischer Scientific) and separated on an in-house packed analytical column (75 µm x 35 cm, particle size 3 µm, Reprosil-

Pur C18, Dr. Maisch) using a gradient from 3% to 80% ACN in 2% formic acid over 80 min. MS scans were performed at 120,000 resolution, m/z range 375-1500. MS/MS analysis was performed in a data-dependent mode, with top speed cycle of 3 s for the most intense multiple-charged precursor ions. Precursor ions were isolated in the quadrupole with a 0.7 m/z isolation window, with dynamic exclusion set to 10 ppm and duration of 45 s. Isolated precursor ions were subjected to collision induced dissociation (CID) at 30 collision energy with a maximum injection time of 50 ms. Produced MS2 fragment ions were detected in the ion trap followed by multinotch (simultaneous) isolation of the ten most abundant fragment ions for further fragmentation (MS3) by higher-energy collision dissociation (HCD) at 55% and detection in the Orbitrap at 50,000 resolutions, m/z range 100-500.

Data analysis was performed using Proteome Discoverer version 2.4 (Thermo Fisher Scientific). The raw file was matched against the Swissprot Homo Sapiens database (March 2020) using Mascot 2.5.1 (Matrix Science) as a database search engine with peptide tolerance of 5 ppm and fragment ion tolerance of 0.6 Da. Tryptic peptides were accepted with one missed cleavage, mono-oxidation on methionine was set as a variable modification, methylthiolation on cysteine and TMTpro-reagent modification on lysine and peptide N-terminus were set as a fixed modification. Peptide-spectrum match (PSM) validation was done with Percolator with the strict false-discovery rate threshold of 1%. TMT Reporter ion intensities were quantified in MS3 spectra at 0.003 Da mass tolerance using the S/N values as abundances without normalization. Only the quantitative results for the unique peptide sequences with the minimum synchronous precursor selection (SPS) match % of 55 and the average S/N above 10 were used for the protein quantification.

ATAC sequencing (extended protocol)

The chromatin accessibility of MLS 1765-92 and EWS TC-71 cells were assessed with ATAC-Seq in triplicates as described (2). Briefly, 50,000 cells were harvested through trypsinization and centrifuged at 500 rcf for 5 min at 4°C. The cell pellet was resuspended in 500 µl cold DPBS, collected by centrifugation and thereafter resuspended in 500 µl cold hypotonic lysis buffer (10 mM Tris-HCl pH 7.5, 10 mM NaCl (Sigma-Aldrich, Merck), 3 mM $MgCl_2$, 0.1% IGEPAL CA-630 (Sigma-Aldrich) and nuclease-free water (UltraPure Distilled water, Invitrogen). Samples were incubated on ice for 15 min and the pellet of intact nuclei was collected after centrifugation at 500 rcf for 10 min at 4°C. DNA was enzymatically tagmented in a 50 µl transposition reaction containing 1x TD buffer, 2.5 µl TDE1 Tagment DNA Enzyme (Illumina) and nuclease-free water during incubation at 37°C for 60 min. A MinElute PCR purification kit (Qiagen) was used to purify transposed DNA, according to the manufacturer's instructions, which was then eluted in 15 µl 10 mM Tris buffer (pH 8, Qiagen). DNA was kept at -20°C until further processing.

Libraries were generated by PCR in a 50 µl reaction containing 1x NEBNext High-Fidelity 2X PCR Master mix (New England Biolabs), 1.25 µM universal primer and 1.25 µM indexing primer (both Integrated DNA Technologies, Supplementary Table 2), 15 µl of the transposed DNA and nuclease-free water at 72°C for 5 min, 98°C for 30 s followed by 10 cycles of amplification at 98°C for 10 s, 63°C for 30 s, 72°C for 1 min and final cooling to 4°C on a T100 Thermal Cycler (Bio-Rad Laboratories). Double-sided bead purification was applied to remove primer dimers and > 1000 base pairs fragments using Agencourt AMPure XP (BD Biosciences). Beads were mixed with sample in a beads-to-sample ratio of 1:2 and incubated at room temperature, first on the bench for 10 min and thereafter on a DynaMag magnet (Thermo Fisher Scientific) for 5 min. The supernatant was then mixed with beads in a beads-to-sample ratio of 1.3:1, followed by 10 min incubation on the bench and 5 min on the magnet. The bead pellet

was washed two times with 200 µl 80% ethanol prepared fresh (Solveco). After drying the beads on the magnet for 10 min, the sample was eluted in 20 µl nuclease-free water. Library quality was assessed with a Fragment Analyzer using a DNF-474 High Sensitivity NGS kit (both Agilent Technologies), according to the manufacturer's instructions. Quantification was performed with quantitative PCR using a KAPA Library Quantification Kit for Illumina platforms (KAPA biosystems). Purified libraries were stored at -20 °C. Libraries were pooled equimolarly and sequenced by National Genomics Infrastructure in Stockholm with a NovaSeq6000 Sequencing System on an Illumina NovaSeq6000 SP flow-cell (Illumina) using paired-end sequencing and a read length of 2x100 base pairs.

ATAC sequencing data analysis

Raw sequencing data were quality checked with MultiQC (v.1.10.1), reads were trimmed from adapter content with TrimGalore (v.0.6.1) and Cutadapt (v.2.3). Unpaired reads were removed (BBMap repair.sh v.38.61b) and reads were thereafter aligned to the reference genome ENSEMBL GRCh38 with Burrows Wheeler Aligner (3). PCR duplicates were eliminated in peak-calling, while mitochondrial reads and ENCODE blacklisted regions (4) were omitted with removeChrom.py and BEDTools intersect. Peak-calling was performed with MACS2 (5) using a false-discovery rate cutoff <0.05 on bam-files sorted based on chromosome coordinates with samtools. Downstream analyses were performed in R (v.4.0.3). The quality of ATAC-Seq data was assessed with ATAC-seqQC (v.1.18.1) (6). The ATAC-Seq peaks displayed the expected characteristic read length distribution for nucleosome-free (<100 base pairs), mononucleosome-bound, dinucleosome-bound etc. fragments and a characteristic peak at or next to the transcription start site for nucleosome-free or nucleosome-bound fragments, respectively. Chromatin regions accessible in at least two out of three replicates were considered open. To assess significantly accessible regions, the R package DiffBind (v.2.16.2) (7) with the DESeq2 algorithm (8) was used with an false-discovery rate cutoff of ≤0.001 and log2 fold change ≥2. Genomic regions were annotated to the closest gene with the annotatePeak function from the R package ChIPSeeker (v.1.24.0) (9) by setting the parameter tssRegion to 1000 base pairs. De novo motif discovery analysis was performed with findMotifsGenome.pl from HOMER (10) using a region size of 200 base pairs.

RNA sequencing (extended protocol)

For RNA analysis, MLS cells (402-91, 1765-92 and 2645-94) were grown on non-coated T25 culture flasks, while EWS cells (TC-71 and 6647) were cultured on collagen-coated (Collagen R, Serva, Thermo Fisher Scientific) flasks, all with cell culture media containing 0.0025% dimethyl sulfoxide (Sigma-Aldrich) for 24h (n=4). Cells were washed with DPBS and collected by scraping in Qiazol lysis reagent (Qiagen). RNA was extracted using miRNeasy micro kit (Qiagen) including DNase treatment, according to the manufacturer's instructions. RNA integrity was assessed with a DNF-471 RNA kit (Agilent technologies) on a Fragment Analyzer. The RNA quality number was >8.3 for all samples. The total RNA concentration was quantified with a Qubit RNA High Sensitivity assay kit on a Qubit 4 Fluorometer (Invitrogen) and then stored at -80°C.

The Smart-seq2 protocol was performed to generate libraries (11) with minor modifications. Reverse transcription was performed in two steps with initial hybridization performed in 6.65 µl, containing 10 ng total RNA, 1 mM dNTP (Sigma-Aldrich) and 1 µM biotinylated adapter sequence oligo-$dT_{30}VN$ (5'-biotin-AAGCAGTGGTATCAACGCAGAGTACT30VN-3', Integrated DNA Technologies). Samples were heated to 72°C for 3 min in a T100 instrument. Then, 1x First-strand buffer (50 mM Tris–HCl pH 8.3,

75 mM KCl and 3 mM $MgCl_2$; Invitrogen), 1 M betaine (Sigma-Aldrich), 5 mM DTT, 10 mM $MgCl_2$, 0.6 µM biotinylated adapter sequence-containing template-switching oligonucleotide (5′-biotin-AAGCAGTGGTATCAACGCAGAGTACAT rGrG+G-3′ with rG = riboguanosine and +G = locked nucleic acid modified guanosine, Eurogentec), 15 U RNaseOUT (Invitrogen), 150 U Superscript II reverse transcriptase (Invitrogen) and nuclease-free water were added to a final reaction volume of 15 µl. Final reaction concentrations are shown. Reverse transcription was performed at 42°C for 90 min, followed by 70°C for 15 min in a T100 instrument. The cDNA was then stored at -20°C.

Preamplification, performed in a 50 µl reaction, containing 1x KAPA HiFi HotStart Ready mix (KAPA Biosystems), 0.1 µM ISPCR primer (5′-AAGCAGTGGTATCAACGCAGAGT-3′, Integrated DNA Technologies) and 6.5 µl cDNA, was first run at 98°C for 3 min, then 15 cycles of 98°C for 20 s, 67°C for 15 s and 72°C for 6 min followed by final incubation at 72°C for 5 min in a T100 instrument. Libraries were purified with Agencourt AMPure XP by mixing beads with sample in a beads-to-sample ratio of 0.8:1, followed by 5 min incubation in room temperature on the bench and additionally 5 min on a DynaMag magnet. Samples were washed twice with 200 µl freshly prepared 80% ethanol. Beads were left to dry on the magnet for 5 min and then resuspended in 17.5 µl nuclease-free water, incubated for 2 min on the bench and additionally 2 min on the magnet before 15 µl of the preamplified cDNA was collected. The cDNA quality was analyzed with a Fragment Analyzer using the DNF-474 High Sensitivity NGS kit. Nextera XT DNA library preparation kit and Nextera XT index Kit v2 (both Illumina) was used to prepare Illumina libraries. Concentrations were assessed with a Qubit 4 Fluorometer using a Qubit dsDNA High Sensitivity Assay Kit (Invitrogen). The 20 µl tagmentation reaction contained 500 pg purified cDNA, 5 µl Amplicon Tagment Mix and 10 µl Tagment DNA buffer. Tagmentation was performed at 55°C for 5 min in a T100 instrument. To stop the reaction, 5 µl Neutralize Tagment buffer was added and incubated for 5 min at room temperature. Then, 15 µl Nextera PCR master Mix and 5 µl of each index adapters were added to the sample in a final reaction volume of 50 µl. Amplification was performed in a T100 instrument at 72°C for 3 min, 95°C for 30 s followed by 16 cycles at 95°C for 10 s, 55°C for 30 s, and 72°C for 30 s, and a final incubation at 72°C for 5 min. Libraries were purified with Agencourt AMPure XP using a beads-to-sample ratio of 0.6:1, as described above. The library concentration was quantified on a Qubit 4 fluorometer with the Qubit dsDNA High Sensitivity Assay Kit. Library quality and fragment size distribution were analyzed with a Fragment Analyzer using the DNF-474 High Sensitivity NGS kit. Libraries were diluted to 10 nM and pooled equimolarly before sequencing on a NextSeq 500 instrument using a NextSeq 500/550 High Output Kit v2.5 (Illumina). Paired-end sequencing with 2x75 base pair reads and 1% PhiX control (Illumina) were used.

RNA sequencing data analysis

Sequencing reads were aligned using STAR (v.2.7.9A) (12) with the ENSEMBL GRCh38 reference genome. HTSeq (v.0.13.5) was applied for read counting (13). Genes with less than an average of three counts were eliminated from downstream analysis. Differential expression analysis was performed by pairwise comparisons between MLS and EWS cell lines using DESeq2 in R (v.4.1.2). Genes were considered significantly regulated if the Benjamini-Hochberg adjusted p-value was <0.05 and fold change ≥2. Only genes with differential expression in all comparisons were used for downstream analysis. Functional enrichment analysis was performed as described in the QMS data analysis section.

Single-cell RNA sequencing and data analysis (extended protocol)

Single-cell RNA sequencing was performed using the PIPseq T2 protocol (Fluent BioSciences) according to the manufacturer's instructions. All reagents were provided by Fluent BioSciences unless otherwise

specified. MLS 402-91 cells were detached using StemPro Accutase (Gibco) and resuspended in cell media. Cells were washed with 1 ml prewarmed cell suspension buffer, then resuspended in 400 µl ice-cold cell suspension buffer and filtered through a 40 µm tip cell strainer (SP Bel-Art) into a 1.5 ml DNA LoBind tube (Eppendorf). Cells were counted with Countess cell counter (Invitrogen) and diluted to 1000 live cells per µl cell suspension buffer with viability at 94% as estimated using trypan blue staining. Low retention tips were used in all steps (Thermo Fisher Scientific).

Particle-templated instant partitions (PIPs) tubes were thawed on ice. The following steps including cell lysis, cDNA synthesis and amplification are done within the PIPs. The cell suspension was mixed by pipetting and 5000 cells in 5 µl was added within the PIP layer. The mixture was mixed 10 times by careful pipetting. Subsequently, 280 µl partitioning reagent was added and the tube was vortexed horizontally at 3000 rpm for 15 s and vertically at 3000 rpm for 2 min in a PIPseq vortex mixer (Fluent BioSciences). In total 230 µl partitioning reagent was removed from the bottom of the tube and discarded, leaving the emulsion intact. Lysis was performed on a PIPseq Dry Bath (Fluent BioSciences) at 66°C for 38 min and at 4°C for 31 min. After lysis, remaining partitioning reagent was removed from the bottom of the tube without disturbing the PIPs. Then, 200 µl room-tempered breaking buffer followed by 40 µl de-partitioning reagent were added on top of the emulsion. The emulsion was broken by inverting the tube three times, vortexing vertically at 3000 rpm for 3 s and inverting the tube three times more. Following a short table centrifugation, all partitioning fluid was removed from the bottom of the tube and discarded, and the remaining sample including PIPs was kept on ice. The PIPs were transferred to 1.5 ml low-retention tubes (Thermo Fisher Scientific) containing 1 ml 1x washing buffer and mixed by flicking, inverting and vortexing, centrifuged for 1 min on a benchtop centrifuge and all washing buffer above the 0.1 ml mark in the tube was removed. Washing was repeated two additional times. The sample was transferred to a 0.2 ml strip tube and briefly centrifuged in a Platefuge plate centrifuge (Benchmark Scientific). With the strip tube in a PIPseq guide rack (Fluent BioSciences), all supernatant above the guide wire was removed, and the sample was stored on ice.

For cDNA synthesis, 33.5 µl RT additive mix, 2 µl 1x TSO, 2.4 µl RT enzyme mix and 1.1 µl nuclease-free water were added to the sample, vortexed and briefly centrifuged. cDNA synthesis was performed on a T-100 instrument (Bio-Rad Laboratories) at 25°C for 30 min, 42°C for 90 min and 85°C for 10 min followed by cooling down to 4°C. After centrifugation, all supernatant above the guide wire was removed and 170 µl 0.5x washing buffer was added. The sample was vortexed, centrifuged and 150 µl supernatant was removed. Washing was repeated two additional times with 150 µl 0.5x washing buffer and all supernatant above the guide wire was removed. The sample was stored at 4°C overnight. For whole transcriptome amplification, 39 µl WTA buffer mix and 0.39 µl WTA primer was added to the sample. After vortexing and centrifugation, amplification was performed on a T-100 instrument at 95°C for 3 min followed by 12 cycles of amplification at 98°C for 15 s and 69°C for 4 min 20 s and thereafter 72°C for 5 min and cooling down to 4°C.

For isolation of cDNA from PIPs, 40 µl IDTE buffer pH 8 was added to each sample and the mixture was added to a 0.45 µm Spin-X Centrifuge Tube Filter (Corning Life Sciences), followed by centrifugation at 13,000 g for 5 min. The flow-through was collected and diluted with IDTE to a final volume of 120 µl. Samples were purified with SPRI beads (Fluent Biosciences) by mixing beads with sample in a beads-to-sample ratio of 0.6:1, followed by 5 min incubation in room temperature on the bench and additionally 5 min on a magnetic tube stand (Fluent BioSciences). Samples were washed twice with 200 µl freshly prepared 85% ethanol (Solveco). Beads were left to dry on the magnet for 5 min and

then resuspended in 22 µl IDTE, incubated for 5 min on the bench and additionally 5 min on the magnet before 20 µl of the preamplified cDNA was collected into a new tube. Concentration was assessed with a Qubit 4 Fluorometer using a Qubit dsDNA High Sensitivity Assay Kit (Invitrogen) and quality control was performed using a D5000 ScreenTape assay on a 4200 TapeStation instrument (both Agilent Technologies), according to the manufacturer's instructions. The sample was stored at -20°C.

For library preparation, 10 ng of amplified and purified cDNA was used. To 40 µl sample, 4 µl library prep buffer and 6 µl library prep enzymes were added, the sample was vortexed, centrifuged and loaded in a T-100 thermocycler at 4°C whereafter fragmentation was performed at 30°C for 8 min, 65°C for 30 min and cooling down to 4°C. For adapter ligation, 5 µl of 3 µM library adapter mix was added to the sample, which was vortexed and centrifuged, and 20 µl library prep mix A was added. The sample was mixed by pipetting, centrifuged and adapter ligation was performed in a T-100 instrument at 20°C for 15 min. The sample was purified using SPRI beads at a beads-to-sample ratio of 0.8:1. The procedure was the same as described above, except elution was performed using 21 µl nuclease-free water of which 20 µl was retrieved. For P7 and P5 indexing PCR, 2.5 µl of each primer was added to the sample, as well as 25 µl library prep mix B. The sample was mixed by pipetting, centrifuged and index PCR was performed on a T-100 instrument at 98°C for 45 s followed by 12 cycles of amplification at 98°C for 15 s, 67°C for 30 s, 69°C for 45 s and a final incubation step at 72°C for 1 min. Purification with double-sided size selection was performed using SPRI beads with an initial beads-to-sample ratio of 0.6:1 on a diluted sample with a volume of 85 µl. Beads were added and mixed by pipetting followed by 7 min incubation in room temperature on the bench and additionally 5 min on a magnetic tube stand. The supernatant was saved and 17 µl SPRI beads were added to reach a beads-to-sample ratio of 0.8:1. After incubation for 5 min at room temperature and 5 min on a magnetic tube stand, the supernatant was discarded, and samples were washed twice with 200 µl freshly prepared 85% ethanol. Beads were left to dry on the magnet for 5 min and then resuspended in 13 µl IDTE, incubated for 5 min on the bench and additionally 5 min on the magnet before 12 µl of the preamplified cDNA was collected into a new tube. Concentration was assessed with a Qubit 4 Fluorometer using a Qubit dsDNA High Sensitivity Assay Kit and quality control was performed using a D5000 ScreenTape assay on a 4200 TapeStation instrument, according to the manufacturer's instructions. The sample was stored at -20°C. The sample was sequenced on a NextSeq 500 instrument using a NextSeq 500/550 High Output Kit v2.5 (Illumina). Paired-end sequencing with 2x75 base pair reads and 15% PhiX control (Illumina) was used.

Preprocessing of data was performed using pipseeker (Fluent BioSciences) generating feature-barcode count matrices for five different sensitivity levels of which sensitivity level 2 was chosen for further analysis. The data was analyzed in R (v.4.3.0) using Seurat (v. 4.4.0) (14). Cells were filtered by unique feature counts, retaining cells with 1500 to 8000 expressed genes. Normalization and scaling of transcript counts was performed using default settings, where the 29 SWI/SNF genes were selected for further analysis. Data visualization was made using Python (v.3.7.3), except for t-distributed Stochastic Neighbor Embedding (t-SNE) plots, which were created in Seurat. The DDIT3 sequence was used for determination of FUS::DDIT3 expression. For analysis of SWI/SNF subtypes, a cell was considered positive for a subtype if at least one subtype-specific component was expressed, e.g., only cBAF had at least one cBAF-specific gene expressed, while cBAF/PBAF had at least one cBAF and one PBAF gene co-expressed.

Glycerol gradient sedimentation assay

Protein complexes were size-separated in a linear glycerol gradient by ultracentrifugation followed by western blot analysis. An 11 ml 10-30% glycerol gradient was generated by underlaying a 30% glycerol buffer (10 mM Tris pH 7.5, 0.1 mM EDTA, 12.5 mM $MgCl_2$, 100 mM KCl, 30% glycerol, supplemented with 1mM DTT and protease inhibitors) below a 10% glycerol buffer (as above but with 10% glycerol) in a capped 14×89 mm ultra-clear centrifuge tube (Beckman Coulter) followed by mixing in a Gradient Master (Gradient Station IP, Biocomp) using the matching default program. Nuclear extracts in 250 mM KCl were collected and diluted to 150 mM salt as described previously (15). Then, 300 µg nuclear extract was diluted to 300 µl with 0% glycerol buffer (as above but with 0% glycerol) and carefully loaded on top of the gradient. Tubes were centrifuged in a SW41 rotor (Optima L-90K Ultracentrifuge, Beckman Coulter) at 4°C for 16 h at 40,000 rpm. Twenty-one fractions (each 0.51 ml) were collected from the top using the Gradient Station (BioComp). The remaining liquid was collected as the 22nd fraction. For western blot analysis, 100 µL of each fraction were mixed with 4x LDS NuPage sample buffer (Thermo Fisher Scientific) and frozen at -20°C.

Whole-cell protein extraction

Whole-cell protein extraction was performed by scraping cells using either DPBS or directly in RIPA lysis buffer (Thermo Fisher Scientific) supplemented with 5 mM EDTA and 1x Halt protease and phosphatase inhibitor cocktail (Both Thermo Fisher Scientific). In the former case, cells were resuspended in RIPA buffer after centrifugation at 4°C for 10 minutes at 450 rcf, and cell lysis was ensured by pipetting the cell suspension during incubation on ice after 5 and 10 minutes. Viscous samples were sonicated for 10 minutes in a BioRuptor (Diagenode), with 30 seconds on/off at 4°C to degrade nucleic acids. Protein extracts were then mixed in 4x NuPAGE LDS sample buffer and frozen at -20°C. Protein concentration measurements were done using the DC protein assay (Bio-Rad).

Western blot

Equal volume of glycerol fractions or equal protein amounts of whole-cell protein extracts were loaded on gels followed by SDS-PAGE using the NuPAGE system (Thermo Fisher Scientific) as previously described (15). Blocked membranes were incubated with primary antibodies overnight at 4°C: 0.2 µg/ml ARID1A (HPA005456, Atlas antibodies), 0.5 µg/ml BAF45D (Ab134942, Abcam), 1.1 µg/ml BAF47 (Ab12167, Abcam), 0.04 µg/ml BAF57 (Ab131328, Abcam), 1 µg/ml BRD4 (Ab128874, Abcam), 1 µg/ml BRD7 (Sc-376180), 0.5 µg/ml BRD9 (Ab137245, Abcam), 0.2 µg/ml BRG1 (sc-17796, Santa Cruz), 0.5 µg/ml BRM (Ab15597, Abcam), 0.7 µg/ml DDIT3 (15204-1, Proteintech, targeting FUS::DDIT3), 0.5 µg/ml EZH2 (#07-689, Merck Millipore), 0.2 µg/ml FUS (sc-47711, Santa Cruz, C-terminal antibody targeting normal FUS), 0.02 µg/ml GAPDH (60004-1-Ig, Proteintech), 0.5 µg/ml GLTSCR1L (HPA029391, Atlas antibodies), 1:30,000 Histone H4 (#04-858, Merck Millipore), 0.2 µg/ml PBRM1 (A301-591A, Bethyl) and 1 µg/ml SS18 (sc-28698, Santa Cruz). Chemiluminescent signals were detected using ImageQuant Amersham 800 (Cytiva) after incubation with SuperSignal West femto Max Sensitivity Substrate or SuperSignal West Dura Extended Duration Substrate (Thermo Scientific).

Immunofluorescence

HT1080 cells were seeded with 30,000 cells per well in 4-well Millicell EZ slides (Merck Millipore) and incubated for 18 h. Cells were then transiently transfected with FUS::DDIT3 in the pEGFP-N1 expression vector using FuGENE 6 Transfection Reagent (Promega) with a 6:1 ratio of transfection reagent (µl) to input DNA (µg), following the manufacturer’s instructions. Transfected cells were fixed 20 h after transfection with 4% formaldehyde (Sigma-Aldrich) for 10 min in room temperature. Cells

were washed once in DPBS before blocking with 1% bovine serum albumin (Sigma-Aldrich) and 0.5% Triton-X100 (Sigma-Aldrich) in DPBS for 20 min. Primary antibody incubation was performed for 3 h at 4°C with antibodies specific for ARID1A (3 µg/ml, HPA005456, Sigma-Aldrich), ARID2 (2 µg/ml, sc-166117, Santa Cruz Biotechnology), BRD4 (4.73 µg/ml, ab128874, Abcam), BRG1 (2 µg/ml, sc-17796, Santa Cruz Biotechnology), CEBPβ (2 µg/ml, sc-150, Santa Cruz Biotechnology), EZH2 (2 µg/ml, #07-689, Merck), FUS (2 µg/ml, sc-47711, Santa Cruz Biotechnology, C-terminal antibody targeting normal FUS), GLTSCR1L (2 µg/ml, HPA029391, Atlas antibodies), JUN (0.235 µg/ml, 9165, Cell Signaling), and STAT3 (0.023 µg/ml, 12640, Cell Signaling). Cells were then incubated with Alexa Fluor 555- or 594-tagged goat anti-mouse or anti-rabbit (Invitrogen), at 4 µg/ml for 2 h. Cells without primary antibody were included as controls. After washing, mounting was performed with ProLong Gold Antifade Mountant (Invitrogen). Z-stack images of stained cells were acquired for EGFP, Alexa 555 and Alexa 594 with Zen Blue (v. 3.6) or Zen Black (v3.0) using a LSM 980 Zeiss microscope and 63X Plan Apo/1.4 oil objective (Zeiss). For co-localization analysis, the FIJI/ImageJ software package (16) was used with the plugin JACoP (17).

Supplementary Figures

Supplementary Figure 1. IP-QMS: Experimental design, quality control and validation

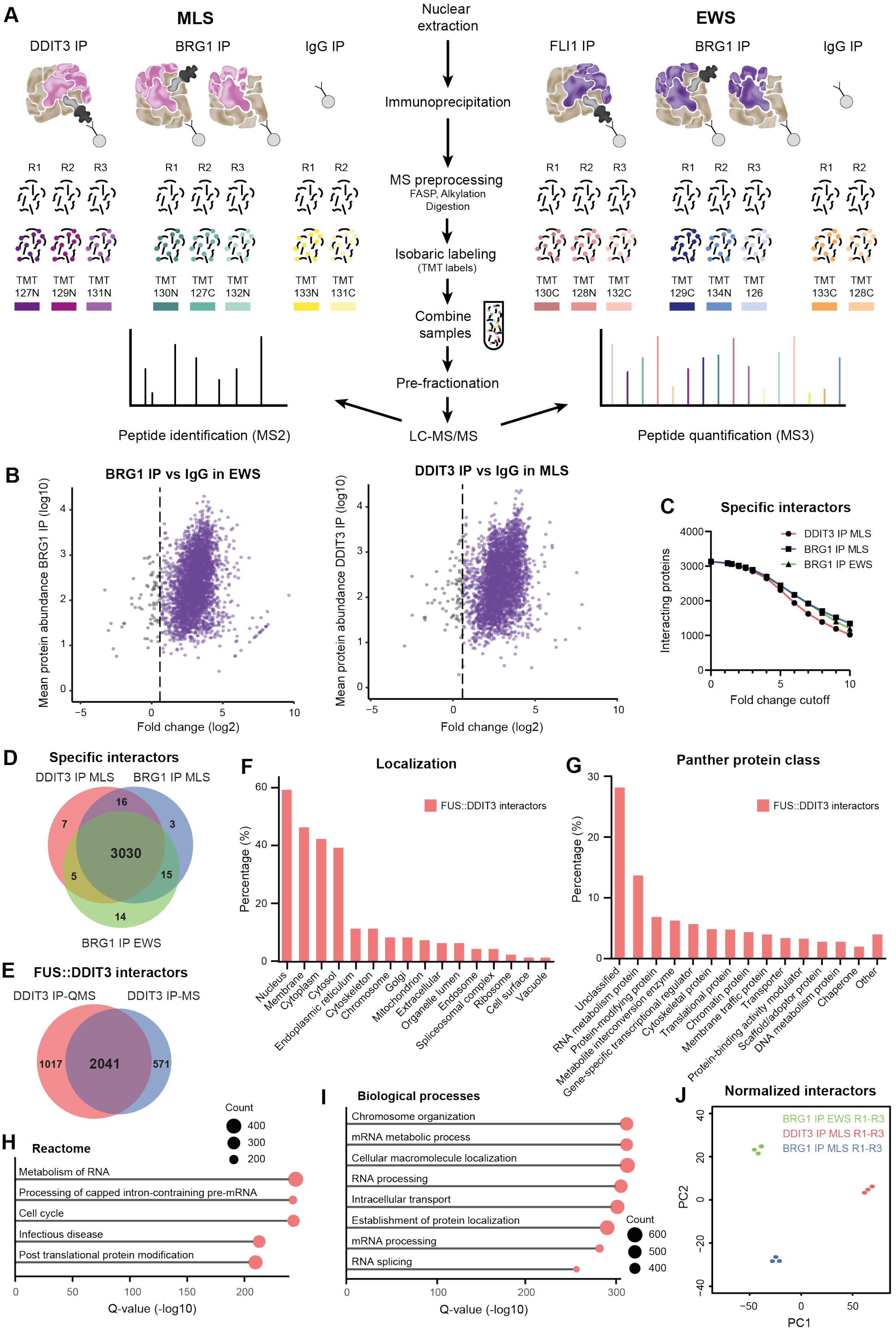

Supplementary Figure 1. IP-QMS: Experimental design, quality control and validation.

A. Experimental design of IP-QMS. FET oncoproteins FUS::DDIT3 (DDIT3 IP) and EWSR1::FLI1 (FLI1 IP), and SWI/SNF complexes (BRG1 IP) were immunoprecipitated using nuclear extracts of MLS 402-91 and EWS TC-71 (n=3, R1-R3). IgG was used as negative control (n=2, R1-R2). Eluates were preprocessed using filter-aided sample preparation (FASP), alkylation and trypsin digestion, followed by isobaric TMT-labeling and LC-MS/MS. TMT-labeled samples were pooled and then pre-fractionated before MS analysis. Peptide identification was performed in MS2 and the abundance of TMT-reporter ions for each sample was quantified in MS3. Note that FLI1 IP samples were excluded from downstream analysis due to too low IP yield.

B. Scatter plots showing the mean abundance of proteins in BRG1 IP versus the enrichment of proteins in BRG1 IP vs IgG in EWS TC-71 and mean abundance of proteins in DDIT3 IP versus the enrichment of proteins in DDIT3 IP vs IgG in MLS 402-91. Each dot indicates a protein (n=3131), where non-enriched (grey) and enriched (purple) proteins are marked, dashed lines indicate the applied fold change cut off: 1.5.

C. Graph depicting the number of specific interactors at different fold change enrichments for each IP compared with their respective IgG control.

D. Venn diagram depicting overlap of specific interaction partners (enriched >1.5 compared to IgG) for DDIT3 IP and BRG1 IP in MLS 402-91 and BRG1 IP in EWS TC-71.

E. Venn diagram depicting overlap of proteins (78%) identified in the DDIT3 IP-QMS analysis with a previous DDIT3 IP-MS experiment (18).

F. Cellular localization of FUS::DDIT3 interaction partners, classified by Proteincenter in the ProteomeDiscoverer software. Note that some proteins belong to several groups.

G. Panther protein class of the FUS::DDIT3 interaction partners. Note that some proteins belong to several groups.

H. Gene sets from Reactome gene set collection enriched for the FUS::DDIT3 interaction partners. The top five most significant gene sets based on q-value are shown. Gene count is indicated by dot size.

I. Gene sets from GO Biological processes gene set collection enriched for the FUS::DDIT3 interaction partners. The top eight most significant gene sets based on q value are shown. Gene count is indicated by dot size.

J. Principal component analysis of IP replicates (R1-R3) for DDIT3 IP and BRG1 IP in MLS 402-91 and BRG1 IP in EWS TC-71 based on protein abundance after normalization to BRG1.

Supplementary Figure 2. Interactions and correlation of transcription factors in MLS and EWS.

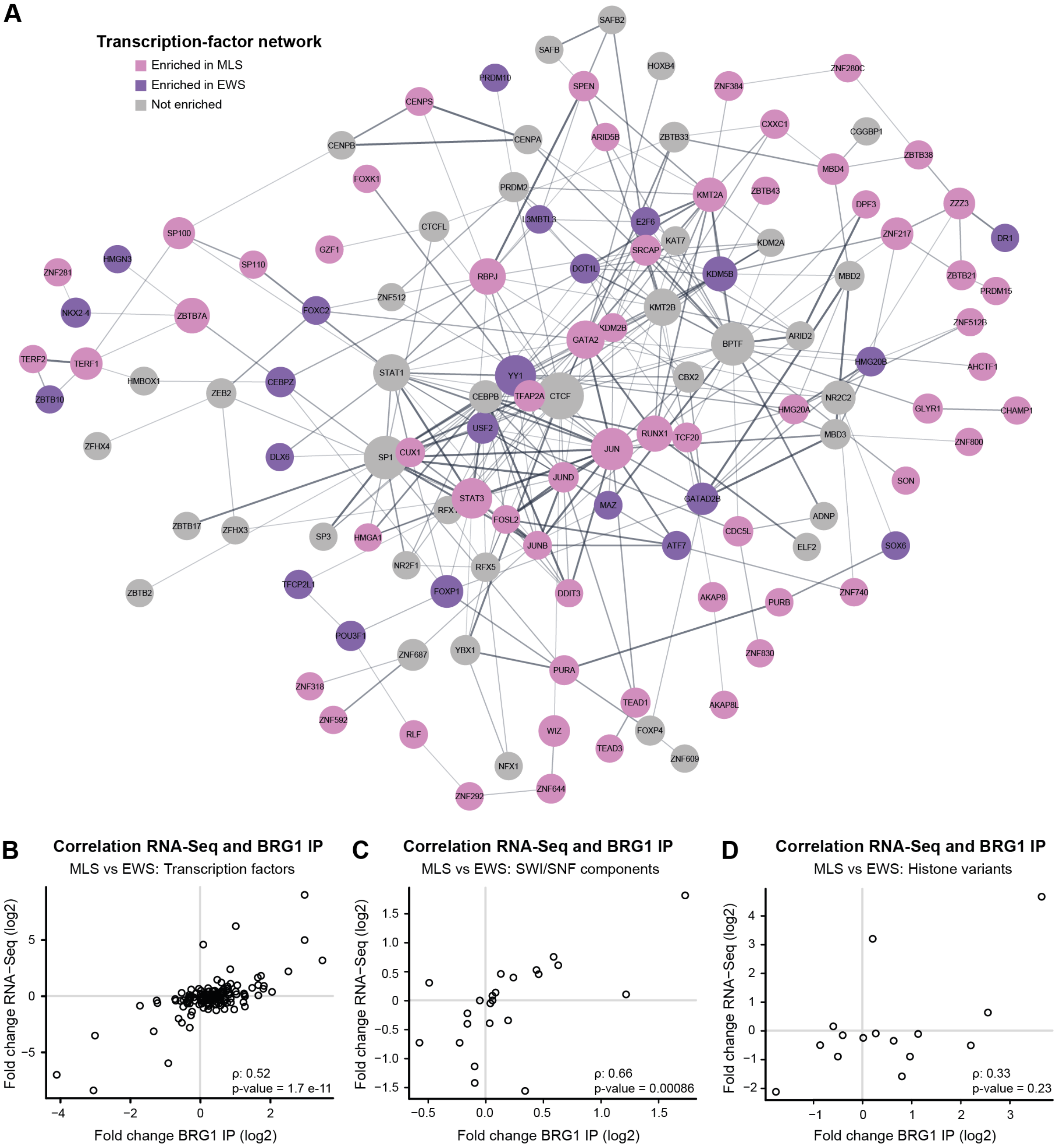

Supplementary Figure 2. Interactions and correlation of transcription factors in MLS and EWS.

A. Network of known protein interactions for transcription factors identified in the IP-QMS data. Transcription factors enriched in BRG1 IP in MLS 402-91 (pink), in EWS TC-71 (purple) or not enriched (grey) are visualized as nodes.

B. Correlation between relative gene expression using bulk RNA-Seq and protein enrichment of transcription factors in BRG1 IP, comparing MLS with EWS. Spearman's correlation coefficient (ρ) is shown, n=139.

C. Correlation between relative gene expression using bulk RNA-Seq and protein enrichment of SWI/SNF components in BRG1 IP, comparing MLS with EWS. Spearman's correlation coefficient (ρ) is shown, n=23.

D. Correlation between relative gene expression using bulk RNA-Seq and protein enrichment histone variants in BRG1 IP, comparing MLS with EWS. Spearman's correlation coefficient (ρ) is shown, n=15.

Supplementary Figure 3. ATAC-Seq and RNA-Seq comparison of MLS and EWS cells.

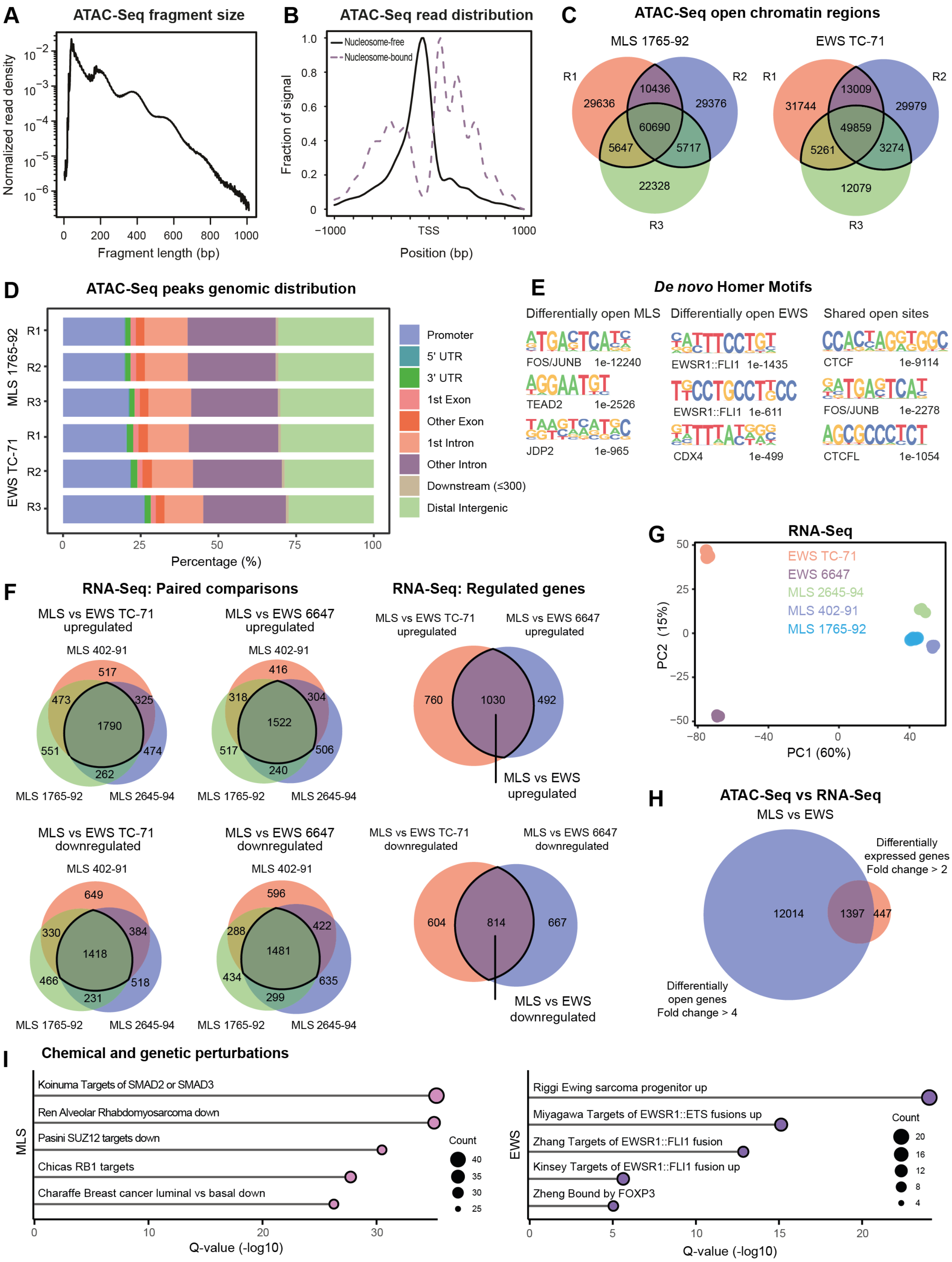

Supplementary Figure 3. ATAC-Seq and RNA-Seq comparison of MLS and EWS cells.

A. Fragment size distribution of ATAC-Seq peaks show normalized read density for different fragment lengths. The ATAC-Seq peaks displayed the expected characteristic read length distribution for nucleosome-free (<100

bp), mononucleosome-bound, dinucleosome-bound etc. fragments every ~200 bp based on the number of nucleosomes bound. One representative example is shown.

B. Distribution of ATAC-Seq signal around transcription start site (TSS) for nucleosome-free (solid line) and nucleosome-bound fragments (dashed line). The ATAC-Seq peaks show a characteristic peak at or next to the transcription start site for nucleosome-free or nucleosome-bound fragments, respectively. One representative example is shown.

C. Venn diagram of overlapping ATAC-Seq open chromatin regions for three replicates (R1-R3) of MLS 1765-92 or EWS TC-71.

D. Genomic distribution of ATAC-Seq peaks with open chromatin regions for three replicates (R1-R3) of MLS 1765-92 and EWS TC-71.

E. Top three enriched de novo motifs from Homer analysis in differentially open sites in MLS, EWS as well as shared open binding sites.

F. Venn diagrams depicting differentially regulated genes using RNA-Seq data (fold change >2) for paired comparisons between the three MLS cell lines 402-91, 1765-92 and 2645-94 and EWS TC-71 or EWS 6647. Overlapping up- or downregulated genes shared in all comparisons were considered regulated between MLS and EWS cells.

G. Principal component analysis of gene expression for three MLS 402-91, 1765-92 and 2645-94 and two EWS TC-71 and 6647 cell lines based on RNA-Seq (n=4, R1-R4).

H. Venn diagram depicting overlap between differentially open genes i.e., genes with a differentially accessible chromatin region (fold change >4) and differentially expressed genes (fold change >2).

I. Enrichment of gene sets from chemical and genetic perturbations with genes that are both upregulated at RNA level and enriched at protein level in BRG1 IP in MLS (n=124) or EWS (N=49). Top five gene sets based on q-value are shown. Gene count is indicated by dot size. Note that no MLS-related gene sets are available in the molecular signature database.

Supplementary Figure 4. Single-cell RNA-Seq analysis.

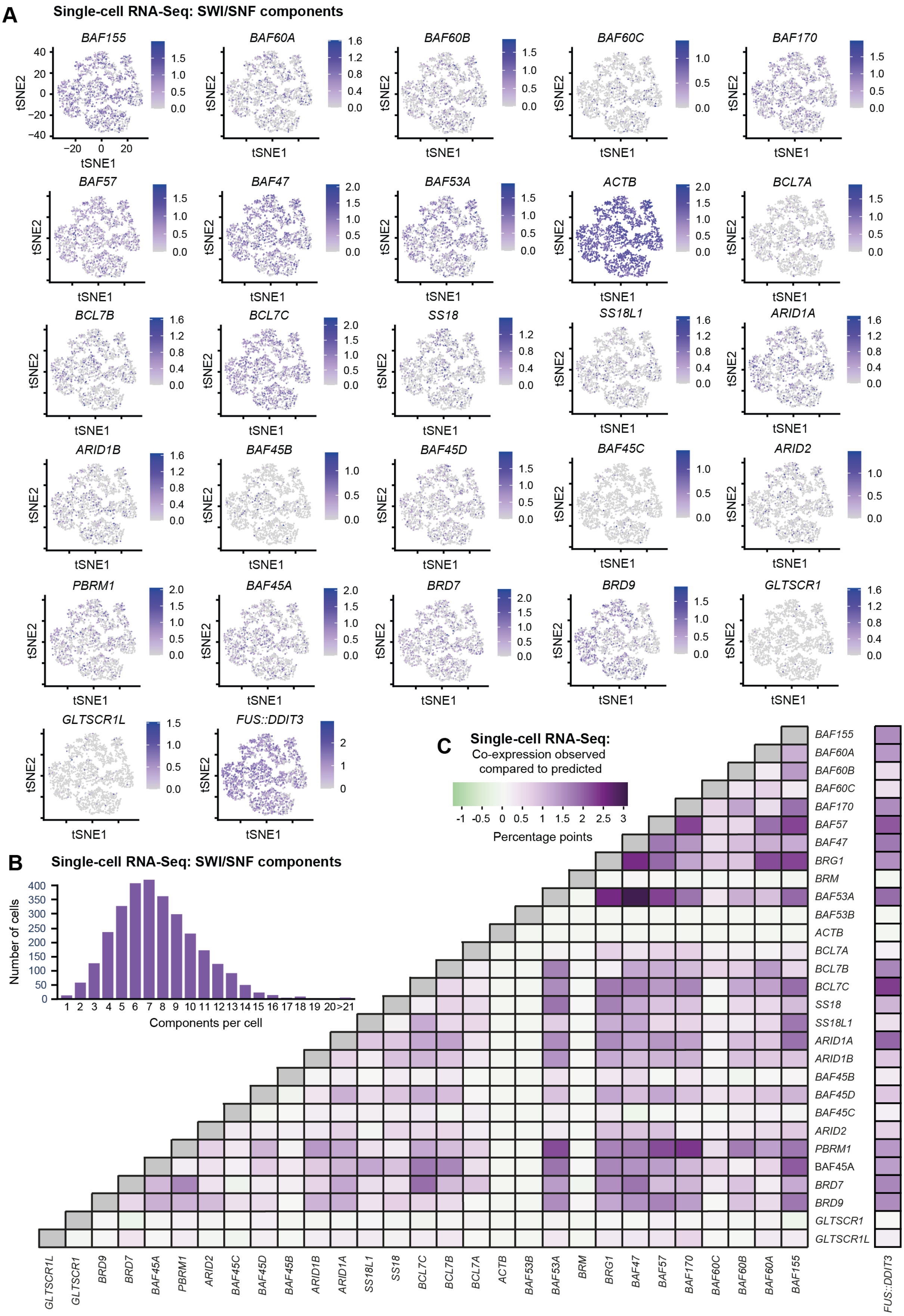

Supplementary Figure 4. Single-cell RNA-Seq analysis.

A. Single-cell expression profiling using single-cell RNA Seq. t-distributed Stochastic Neighbor Embedding (t-SNE) plots based on the whole transcriptome showing the expression of SWI/SNF components and FUS::DDIT3 in MLS 402-91 cells (n=2923 cells).

B. Number of SWI/SNF components expressed in each cell based on single-cell RNA-Seq data (n=2923 cells).

C. Heatmap visualizing the difference between observed co-expression of SWI/SNF components as well as *FUS::DDIT3,* compared to their predicted likelihood of co-expression by chance. For example, *BAF47* was expressed in 56% of the cells and *BAF53A* in 46% of the cells. Consequently, 25.8% of the cells were expected to express both genes if their expression were not dependent on each other. Here, the observed co-expression was 29.0%, resulting in 3.2 percentage points higher (purple) co-expression than expected.

Supplementary Figure 5. Complete western blot membranes.

A **Complete western blots for Figure 4C: SWI/SNF components**

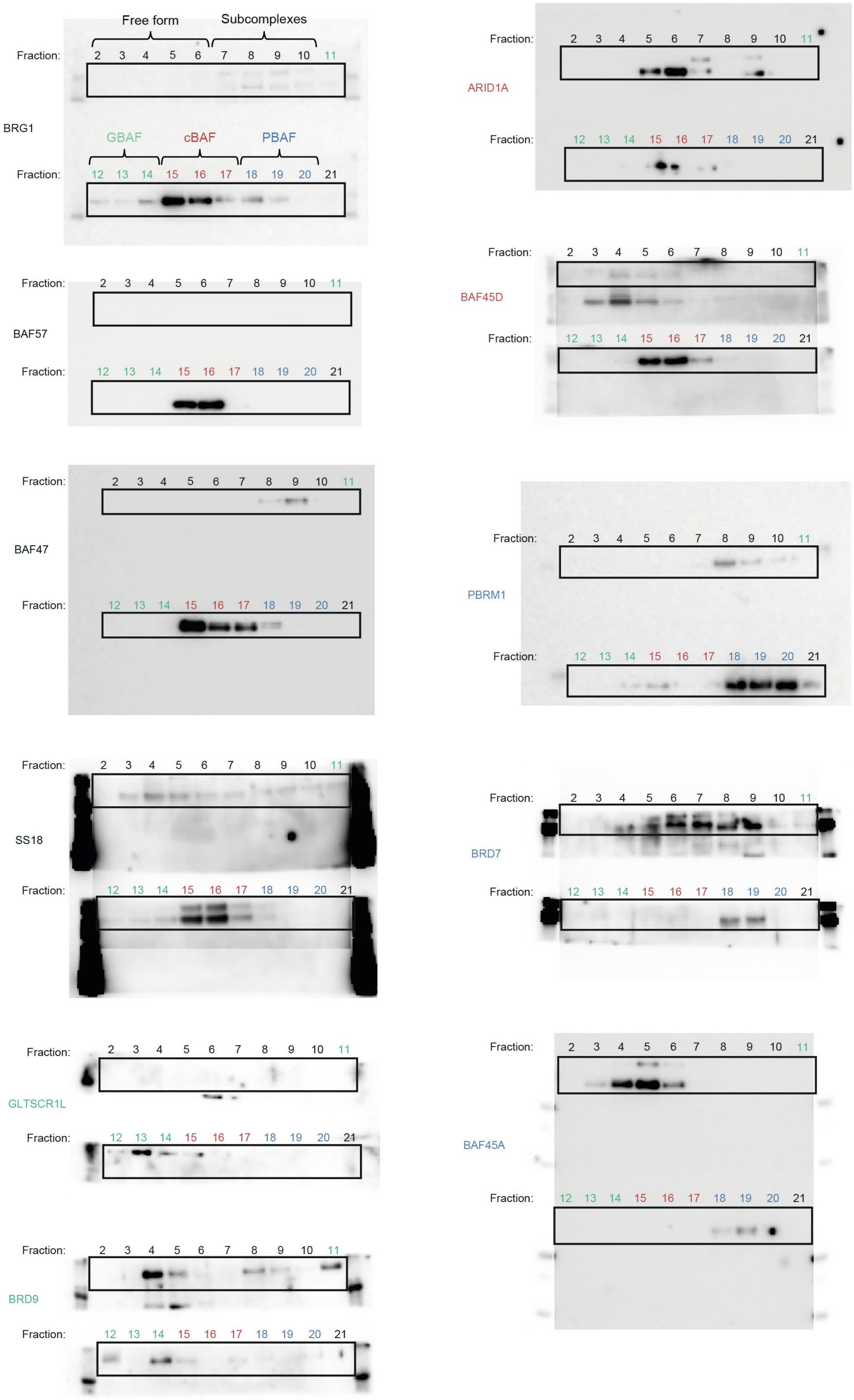

B **Complete western blots for Figure 4C: Interaction partners**

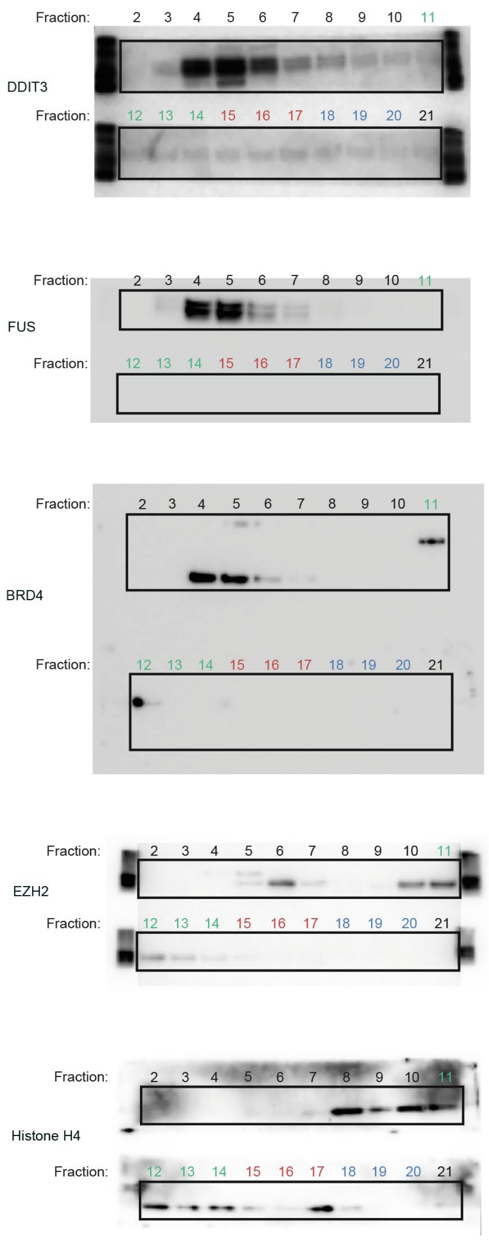

C **Complete western blots for Figure 4G**

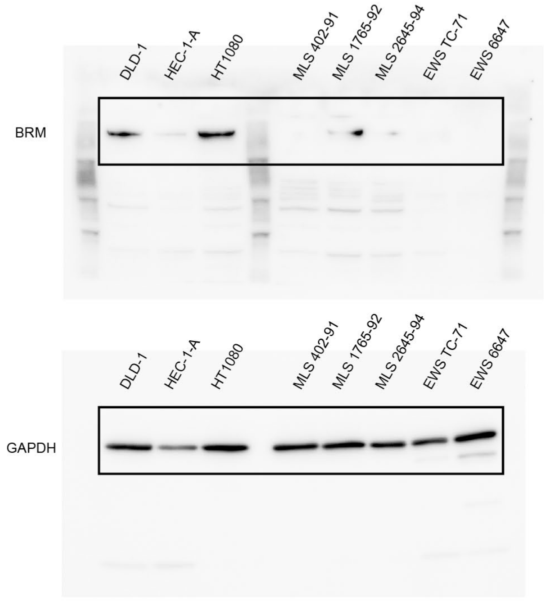

Supplementary Figure 5. Complete western blot membranes.

A. Complete western blot membranes for the glycerol gradient sedimentation assay in Figure 4C, visualizing SWI/SNF components in fractions 2 to 21. For each antibody, the two membrane pieces were treated the same, both during imaging and enhancement of contrast. Note that the concentration of protein in each fraction is low.

B. Complete western blot membranes for the glycerol gradient sedimentation assay in Figure 4C, visualizing interaction partners in fractions 2 to 21. For each antibody, the two membrane pieces were treated the same, both during imaging and enhancement of contrast. Note that the concentration of protein in each fraction is low.

C. Complete western blot membranes for Figure 4G visualizing BRM expression in whole-cell extracts of indicated cell lines. GAPDH was used as loading control. For each antibody, all samples were treated the same, both during imaging and enhancement of contrast.

Supplementary Table legends

List and description of Supplementary Tables. A, B, C etc. depict different Table worksheets.

Supplementary Table 1. QMS raw data

Supplementary Table 2. ATAC-Seq primers

Supplementary Table 3. Specific interactors IP vs IgG

A. Immunoprecipitated proteins in BRG1 IP vs IgG in MLS 402 cells.
B. Immunoprecipitated proteins in BRG1 IP vs IgG in EWS TC-71 cells.
C. Immunoprecipitated proteins in DDIT3 IP vs IgG in MLS 402 cells.

Supplementary Table 4. Characterization and enrichment FUS::DDIT3 interactors

A. Localization
B. Panther protein class
C. GO Biological processes gene sets
D. Reactome gene sets

Supplementary Table 5. Proteins analyzed in comparison between BRG1 IP MLS and EWS (comparison 1)

A. All proteins
B. SWI/SNF components and selected interaction partners

Supplementary Table 6. BRG1 interaction partners

A. Enrichment of BRG1 interactors in comparison between BRG1 IP MLS and EWS (comparison 1)
B. Enrichment of BRG1 interactors in comparison between DDIT3 IP and BRG1 IP in MLS (comparison 2)

Supplementary Table 7. Transcription factors

A. Enrichment of transcription factors in comparison between BRG1 IP MLS and EWS (comparison 1)
B. Enrichment of transcription factors in comparison between DDIT3 IP and BRG1 IP in MLS (comparison 2)

Supplementary Table 8. Histone variants

A. Enrichment of histone variants in comparison between BRG1 IP MLS and EWS (comparison 1)
B. Enrichment of histone variants in comparison between DDIT3 IP and BRG1 IP in MLS (comparison 2)
C. Histone variants in Nextprot

Supplementary Table 9. Differentially open motifs

A. Homer de novo Motif results MLS differentially open sites
B. Homer de novo Motif results EWS differentially open sites
C. Homer de novo Motif Results shared open sites

Supplementary Table 10. Differentially expressed genes between MLS and EWS

Supplementary Table 11. ATAC- and RNA-Seq data

A. Genes differentially expressed and differentially open between MLS and EWS
B. Genes differentially upregulated and differentially more open between MLS and EWS
C. Genes differentially downregulated and differentially more open between MLS and EWS
D. Genes differentially upregulated and differentially less open between MLS and EWS
E. Genes differentially downregulated and differentially less open between MLS and EWS

Supplementary Table 12. Overlap of regulated genes and enriched proteins in BRG1 IP in MLS vs EWS

Supplementary Table 13. Enrichment, characterization and function of FET-sarcoma-specific transcription factors

Supplementary Table 14. Proteins analyzed in comparison between DDIT3 IP and BRG1 IP MLS (comparison 2)

A. All proteins
B. SWI/SNF components and selected interaction partners

Supplementary Table 15. Single-cell RNA-Seq data

Supplementary Table 16. Single-cell SWI/SNF gene co-expression

References

1. Wiśniewski JR, Zougman A, Nagaraj N, Mann M. Universal sample preparation method for proteome analysis. Nat Methods. 2009;6(5):359-62.

2. Buenrostro JD, Giresi PG, Zaba LC, Chang HY, Greenleaf WJ. Transposition of native chromatin for fast and sensitive epigenomic profiling of open chromatin, DNA-binding proteins and nucleosome position. Nat Methods. 2013;10(12):1213-8.

3. Li H. Aligning sequence reads, clone sequences and assembly contigs with BWA-MEM. arXiv. 2013:1303.3997.

4. Amemiya HM, Kundaje A, Boyle AP. The ENCODE Blacklist: Identification of Problematic Regions of the Genome. Sci Rep. 2019;9(1):9354.

5. Zhang Y, Liu T, Meyer CA, Eeckhoute J, Johnson DS, Bernstein BE, et al. Model-based analysis of ChIP-Seq (MACS). Genome Biol. 2008;9(9):R137.

6. Ou J, Liu H, Yu J, Kelliher MA, Castilla LH, Lawson ND, et al. ATACseqQC: a Bioconductor package for post-alignment quality assessment of ATAC-seq data. BMC Genomics. 2018;19(1):169.

7. Ross-Innes CS, Stark R, Teschendorff AE, Holmes KA, Ali HR, Dunning MJ, et al. Differential oestrogen receptor binding is associated with clinical outcome in breast cancer. Nature. 2012;481(7381):389-93.

8. Love MI, Huber W, Anders S. Moderated estimation of fold change and dispersion for RNA-seq data with DESeq2. Genome Biol. 2014;15(12).

9. Yu G, Wang LG, He QY. ChIPseeker: an R/Bioconductor package for ChIP peak annotation, comparison and visualization. Bioinformatics. 2015;31(14):2382-3.

10. Heinz S, Benner C, Spann N, Bertolino E, Lin YC, Laslo P, et al. Simple combinations of lineage-determining transcription factors prime cis-regulatory elements required for macrophage and B cell identities. Mol Cell. 2010;38(4):576-89.

11. Picelli S, Björklund Å K, Faridani OR, Sagasser S, Winberg G, Sandberg R. Smart-seq2 for sensitive full-length transcriptome profiling in single cells. Nat Methods. 2013;10(11):1096-8.

12. Dobin A, Davis CA, Schlesinger F, Drenkow J, Zaleski C, Jha S, et al. STAR: Ultrafast universal RNA-seq aligner. Bioinformatics. 2013;29(1):15-21.

13. Anders S, Pyl PT, Huber W. HTSeq-A Python framework to work with high-throughput sequencing data. Bioinformatics. 2015;31(2):166-9.

14. Hao Y, Hao S, Andersen-Nissen E, Mauck WM, 3rd, Zheng S, Butler A, et al. Integrated analysis of multimodal single-cell data. Cell. 2021;184(13):3573-87.e29.

15. Lindén M, Vannas C, Österlund T, Andersson L, Osman A, Escobar M, et al. FET fusion oncoproteins interact with BRD4 and SWI/SNF chromatin remodeling complex subtypes in sarcoma. Mol Oncol. 2022.

16. Schindelin J, Arganda-Carreras I, Frise E, Kaynig V, Longair M, Pietzsch T, et al. Fiji: an open-source platform for biological-image analysis. Nat Methods. 2012;9(7):676-82.

17. Bolte S, CordeliÈRes FP. A guided tour into subcellular colocalization analysis in light microscopy. J Microsc. 2006;224(3):213-32.

18. Lindén M, Thomsen C, Grundevik P, Jonasson E, Andersson D, Runnberg R, et al. FET family fusion oncoproteins target the SWI/SNF chromatin remodeling complex. EMBO Rep. 2019;20(5).
